# Polyphosphinoborane Block Copolymer Synthesis Using Catalytic Reversible Chain‐Transfer Dehydropolymerization

**DOI:** 10.1002/anie.202216106

**Published:** 2022-12-08

**Authors:** James J. Race, Alex Heyam, Matthew A. Wiebe, J. Diego‐Garcia Hernandez, Charlotte E. Ellis, Shixing Lei, Ian Manners, Andrew S. Weller

**Affiliations:** ^1^ Department of Chemistry University of York Heslington York YO10 5DD UK; ^2^ Chemistry Research Laboratories University of Oxford Oxford OX1 3TA UK; ^3^ Department of Chemistry University of Victoria Victoria BC, V8P 5C2 Canada; ^4^ Centre for Advanced Materials and Related Technology (CAMTEC) University of Victoria Victoria BC, V8P 5C2 Canada

**Keywords:** Block Copolymer, Borane, Dehydropolymerization, Phosphine, Self-Assembly

## Abstract

An amphiphilic block copolymer of polyphosphinoborane has been prepared by a mechanism‐led strategy of the sequential catalytic dehydropolymerization of precursor monomers, H_3_B ⋅ PRH_2_ (R=Ph, n‐hexyl), using the simple pre‐catalyst [Rh(Ph_2_PCH_2_CH_2_PPh_2_)_2_]Cl. Speciation, mechanism and polymer chain growth studies support a step‐growth process where reversible chain transfer occurs, i.e. H_3_B ⋅ PRH_2_/oligomer/polymer can all coordinate with, and be activated by, the catalyst. Block copolymer [H_2_BPPhH]_110_‐*b*‐[H_2_BP(n‐hexyl)H]_11_ can be synthesized and self‐assembles in solution to form either rod‐like micelles or vesicles depending on solvent polarity.

## Graphical abstract

Amphiphilic block copolymers of polyphosphinoboranes, [H_2_BPPhH]_110_‐*b*‐[H_2_BP(n‐hexyl)H]_11_, have been prepared by a mechanism‐led strategy of the catalytic reversible chain transfer dehydropolymerization of precursor monomers, H_3_B ⋅ PRH_2_ (R=Ph, h‐hexyl). These self‐assemble in THF:hexane solutions to form nanostructured rod‐like micelles, the morphology of which can be controlled by solvent polarity.

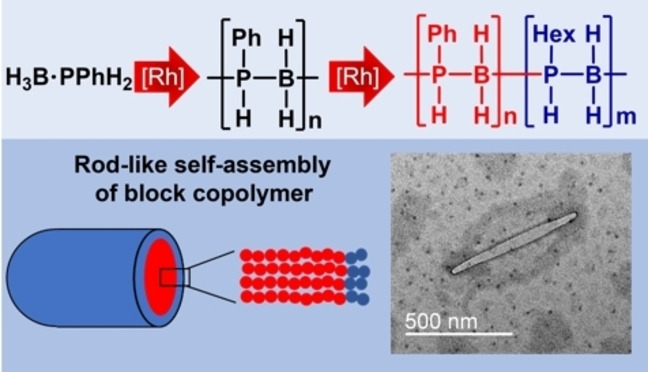

## Introduction

**Scheme 1:**
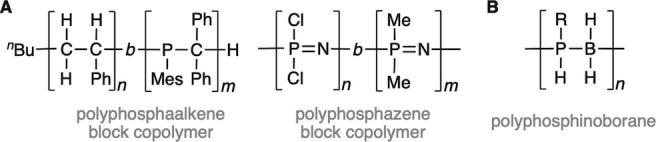
A) Examples of block copolymers of polyphosphaalkanes^[12]^ and polyphosphazenes.^[13]^ B) Polyphosphinoborane (R=alkyl, aryl).

Organic block copolymers (BCPs), in which chemically‐distinct segments of monomer units are linked together in a polymer chain, have played an important role in the development of macromolecular science.[^1^, ^2^, ^3^, ^4^] The solid‐state and solution self‐assembly behavior of BCPs has led to technologically‐important ordered nanostructures,^[5]^ such as films containing phase‐separated nanodomains and micelles. Self‐assembled BCPs have found a variety of applications which include nanolithography, high‐performance additives, and delivery agents in biomedicine.^[6]^ The synthesis of BCPs based on inorganic elements is also of substantial interest as a result of the complementary functionality that can be introduced, such as plasma etch‐resistance, redox‐activity, and useful preceramic properties. To date, studies have mainly been limited to a small group of polymers such as polysiloxanes,[^7^, ^8^, ^9^] and polyferrocenylsilanes[^10^, ^11^] in addition to several other phosphorus‐containing systems, including polyphosphaalkenes^[12]^ and polyphosphazenes,[^13^, ^14^] (Scheme 1A).

Polyphosphinoboranes [H_2_BPRH]_n_ (R=aryl, alkyl, Scheme 1B), have attracted particular recent attention due to the valence‐isoelectronic relationship they have with polyolefins; as well as their potential to act as lithographic resists,^[15]^ precursors to PB ceramic materials,[^16^, ^17^] hydrophilic surfaces,^[18]^ swellable gels,^[19]^ and flame‐retardants.^[20]^ BCPs based on polyphosphinoboranes are currently unknown. These materials would be of interest for the fabrication of nanostructured thin‐films and self‐assembled micelles in solution where the characteristics of the phosphinoborane segment may be exploited in nanopatterning and other materials applications.

**Scheme 2:**
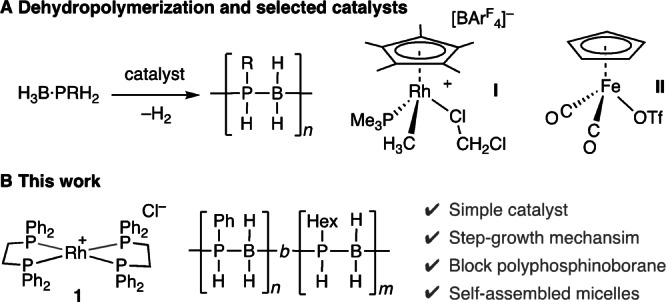
A) Phosphine–borane dehydropolymerization; B) This work.

Polyphosphinoborane homopolymers are generally (but not exclusively[^21^, ^22^, ^23^, ^24^]) synthesized by the catalytic dehydropolymerization[^25^, ^26^] of precursor primary phosphine–boranes, H_3_B ⋅ PRH_2_, where H_2_ is the only by‐product (Scheme 2A). While initial reports used melt conditions at temperatures of up to 130 °C with catalysts such as [Rh(COD)Cl]_2_,^[27]^ more recently catalyst systems that operate in solution at lower temperatures (e.g. arenes, 100 °C) have been reported,[^28^, ^29^, ^30^, ^31^] exemplified by [Cp*RhCH_3_(PMe_3_)(ClCH_2_Cl)][BAr^F^_4_], **I**,^[32]^ and versatile CpFe(CO)_2_OTf, **II**.[^18^, ^33^, ^34^] A step‐growth‐like mechanism is suggested to operate using catalyst **I**.^[32]^ In contrast, for **II** a non‐living chain‐growth mechanism is proposed.^[33]^ Such catalyst‐dependent changes in mechanism has been noted before for group 13/15 dehydropolymerizations.[^30^, ^35^, ^36^] We now report, by using the simple pre‐catalyst [Rh(Ph_2_PCH_2_CH_2_PPh_2_)_2_]Cl, **1**, and harnessing a step‐growth like mechanism in which reversible chain transfer also occurs,^[36]^ a polyphosphinoborane block copolymer can be prepared containing aromatic and alkyl substituted segments. We also describe that this material shows sufficient amphiphilicity to self‐assemble in solution to form rod‐like micelle nanostructures, Scheme 2B.

## Results and Discussion

We have previously briefly reported that the Rh^I^ cationic catalyst, [Rh(L^1^)(η^6^‐FC_6_H_5_)[BAr^F^_4_] (L^1^=Ph_2_PCH_2_CH_2_CH_2_PPh_2_) will dehydropolymerize H_3_B ⋅ PPhH_2_ under melt conditions to form [H_2_BPPhH]_n_, while solution conditions produced shorter‐chain oligomers.^[37]^ Detailed stoichiometric reactivity studies using this catalyst, with primary and secondary phosphine–boranes, show that a Rh^III^ complex with a chelating P−H activated diboraphosphine is a plausible intermediate, e.g. [Rh(L^1^)H(σ,η‐PR_2_ ⋅ BH_2_PR_2_ ⋅ BH_3_)][BAr^F^_4_], **III** (R=H, Ph, 3,5‐(CF_3_)_2_C_6_H_3_);[^37^, ^38^] Wanting to use a simpler, potentially more robust, catalyst that did not involve the costly [BAr^F^_4_]^−^ anion we explored the use of air‐stable [Rh(Ph_2_PCH_2_CH_2_PPh_2_)_2_]Cl, **1**, in the anticipation that the chelating phosphine may become labile under the conditions of catalysis (≈100 °C). Optimization of conditions showed that 1 mol% **1**, 1.25 M H_3_B ⋅ PPhH_2_, heated at 100 °C in toluene for 19 hours in a sealed thick‐walled NMR tube, followed by precipitation into hexanes, afforded [H_2_BPPhH]_n_ as a white solid in 86 % isolated yield.^[39]^ GPC analysis (relative to polystyrene standards, see later) showed an essentially mono‐modal distribution: *M*_n_=26 500 g mol^−1^, *Ð*=1.6 (Table 1). NMR spectroscopic data are fully consistent with those reported previously for [H_2_BPPhH]_n_,[^27^, ^33^] in particular in the ^31^P NMR spectrum a single environment is observed at δ −49.4 as a broad doublet, ^*1*^ *J*(PH)=355 Hz. Under these conditions no significant amounts of shorter chain, or cyclic, oligomers are observed.[^27^, ^40^] Complex **1** will also dehydropolymerize relatively electron‐poor H_3_B ⋅ P(3,5‐(CF_3_)_2_C_6_H_3_)H_2_ to form higher molecular weight polyphosphinoborane [H_2_BP(3,5‐(CF_3_)_2_C_6_H_3_)H]_n_, *M*_n_=127 500 g mol^−1^, *Ð*=1.2 in the same reaction time (Figure S6).^[18]^ Here, the atacticity of the polymer backbone is revealed in the ^31^P NMR spectrum by a statistical distribution of proposed rr, [rm, mr] or mm triads, δ(^31^P{^1^H}) −45.6, −46.9, −48.3 (1 : 2 : 1 respectively), as noted previously.[^18^, ^23^] n‐Hexyl phosphine—borane is also dehydropolymerized using **1** to form [H_2_BP(n‐hexyl)H]_n_ as a colorless, hexane‐soluble, oil, *M*_n_=33 000 g mol^−1^, *Ð*=1.3;[^17^, ^34^] but in this case a higher catalyst loading (3 mol%) and longer reaction times (66 hours) are required, consistent with the lower reactivity of alkyl phosphines.^[16]^ Purification of this hexane‐soluble polymer was by passage through a short silica plug using CH_2_Cl_2_, although this does not remove a trace of (H_3_B)_2_ ⋅ dppe (see later). In the ^31^P NMR spectrum a broad signal at δ −63.0 is observed.[^17^, ^34^] GPC analysis also showed a small amount of lower molecular weight oligomers (*M*_n_ ≈2 500 g mol^−1^).

**Table 1:** Phosphine–borane dehydropolymerization.

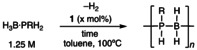					
R	mol %	*M*_n_^[a]^	*Ð*	Yield^[b]^	Time [hrs]
Ph	1	26 500	1.6	86 %	19
3,5‐(CF_3_)_2_C_6_H_3_	1	127 500	1.2	62 %	19
n‐hexyl	3	33 000	1.3	50 % ^[c]^	66

[a] Relative to polystyrene standards, THF (0.1 w/w% [N^n^Bu_4_]Br), 2.0 mg ml^−1^. [b] Isolated yield, [c] A persistent, but small, amount of co‐product is also formed, which is tentatively assigned as cyclic oligomers.

**Scheme 3:**
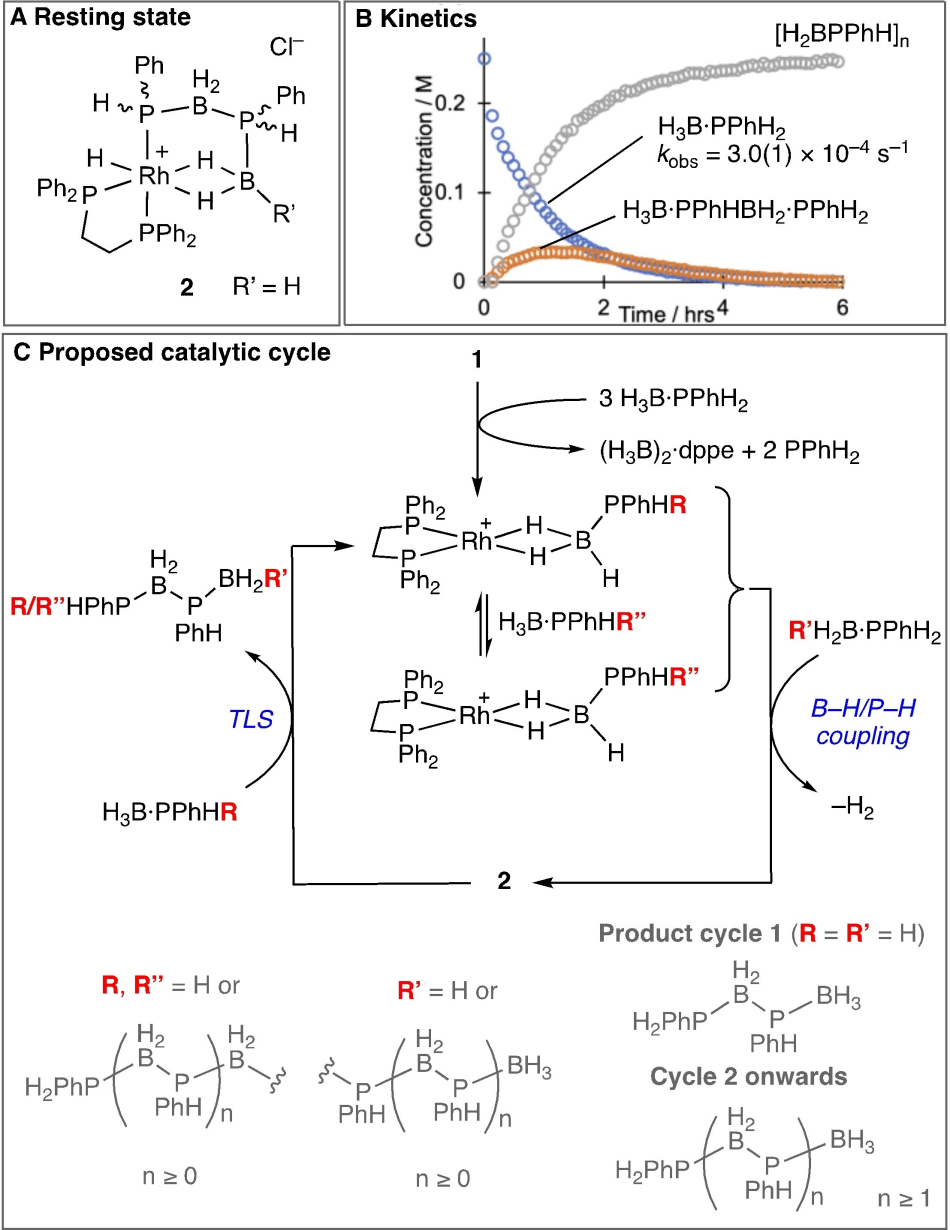
A) Suggested resting state at the early stages of catalysis; B) Temporal evolution of catalysis (3 mol% **1**, 0.25 M H_3_B ⋅ PPhH_2_); C) Proposed, simplified, catalytic cycle and P−B coupled products.

In situ catalyst speciation studies (3 mol% **1**, 0.25 M H_3_B ⋅ PRH_2_) using ^31^P{^1^H} NMR spectroscopy showed that in all cases (H_3_B)_2_ ⋅ dppe is formed immediately (δ 18.7^[41]^) alongside free PRH_2_, demonstrating loss of dppe in the active catalyst. Focusing on H_3_B ⋅ PPhH_2_ in the early stages of catalysis (10 mins, 23 % conversion of monomer) ^1^H NMR spectroscopic analysis of the hydride region showed broad environments centered at δ −1.9 and δ −14.2 in a 3 : 1 relative integral respectively. Repeating at 10 mol% **1** for improved signal intensity, revealed more than one resonance in each chemical shift range, and that only the relative integral 3 H signal sharpened on decoupling ^11^B, identifying these signals as being due to Rh⋅⋅⋅H_3_B and Rh−H respectively.^[42]^ In addition to free (poly)phosphinoboranes, the ^11^B NMR spectrum displayed signals at δ −3.1 and δ −16.6. The ^31^P{^1^H} NMR spectrum was uninformative with regard to identifying organometallic species due broad and weak signals that also overlap with polymer/oligomer signals. These data are similar to those reported for diboraphosphine complexes **III** [R_2_=Ph_2_, CyH; e.g. δ(^11^B) 3.2, −27.2, δ(^1^H) −1.19, −14.10],[^37^, ^38^] and the multiple environments observed are consistent with different diastereoisomers,^[37]^ i.e., [Rh(Ph_2_PCH_2_CH_2_PPh_2_)H(σ,η‐PPhH ⋅ BH_2_PPhH ⋅ BH_3_)]Cl, **2**, Scheme 3A. Complex **2**, and a similar mixture of diastereoisomers, can also be formed by direct addition of a slight excess of H_3_B ⋅ PPhHBH_2_ ⋅ PPhH_2_ ^[32]^ to [Rh(Ph_2_PCH_2_CH_2_PPh_2_)(η^6^‐C_6_H_5_F)][BAr^F^_4_]^[43]^ at 298 K in d_8_‐toluene. ESI‐MS analysis^[44]^ showed a molecular ion at *m*/*z*=747.2 (calc. 747.2) with the correct isotopologue pattern. Irrespective of the method of synthesis, accompanying **2** are unidentified minor hydride signals observed between −15 to −16 ppm, that become dominant as catalysis reaches completion. These are currently unidentified,^[45]^ but as catalysis restarts on addition of more H_3_B ⋅ PPhH_2_ they are not deactivation products. Complex **2**, at least at the early stages of catalysis, is thus identified as a likely resting state.

Following reaction progress by ^1^H NMR spectroscopy at 100 °C (3 mol% **1**), using the distinctive P−H resonances between 5.5 and 3.5 ppm, reveals a first order decay of H_3_B ⋅ PPhH_2_ (0.25 M, *k*_obs_=3.0(1)×10^−4^ s^−1^), linear diboraphosphine H_3_B ⋅ PPhHBH_2_ ⋅ PPhH_2_ as an intermediate and the growth in of oligomeric/polymeric [H_2_BPPhH]_n_, Scheme 3B. Recharging with H_3_B ⋅ PPhH_2_ restarts catalysis (*k*_obs_=1.5(1)×10^−4^ s^−1^). In a separate experiment (3 mol% **1**) using H_3_B ⋅ PPhHBH_2_ ⋅ PPhH_2_ (0.14 M) as the precursor resulted in its clean first order consumption (*k*_obs_=1.0(1)×10^−4^ s^−1^) to form [H_2_BPPhH]_n_ (*M*_n_=61 000 g mol^−1^, *Ð*=1.3^[40]^) with no H_3_B ⋅ PPhH_2_ observed to the detection limit of ^1^H NMR spectroscopy, indicating that depolymerization to H_3_B ⋅ PPhH_2_ is not significant (Figure S23). Combined these observations on the resting state and the temporal evolution of substrates and products lead us to propose a simplified mechanism, Scheme 3C, which is based on previous, detailed, mechanistic studies using the closely related dehydrocoupling of secondary phosphine–boranes.^[38]^ The essential components of this are: *(i)* the P/B dehydrocoupling of −PPhH_2_ and −BH_3_ end groups at the metal center to form **2** (R′=H), or a close analog thereof, i.e. R′=[PPhHBH_2_]_n_PPhH ⋅ BH_3_; *(ii)* a reversible chain transfer between bound and free phosphine–boranes; *(iii)* a turnover limiting step that involves substitution of the newly coupled oligomer with another phosphine–borane (monomer/oligomer/polymer), that leads to the observed first‐order decay of monomers H_3_B ⋅ PPhH_2_ or H_3_B ⋅ PPhHBH_2_ ⋅ PPhH_2_; *(iv)* an overall step‐growth like mechanism that supports the observation of H_3_B ⋅ PPhHBH_2_ ⋅ PPhH_2_ as a persistent intermediate.

**Scheme 4:**
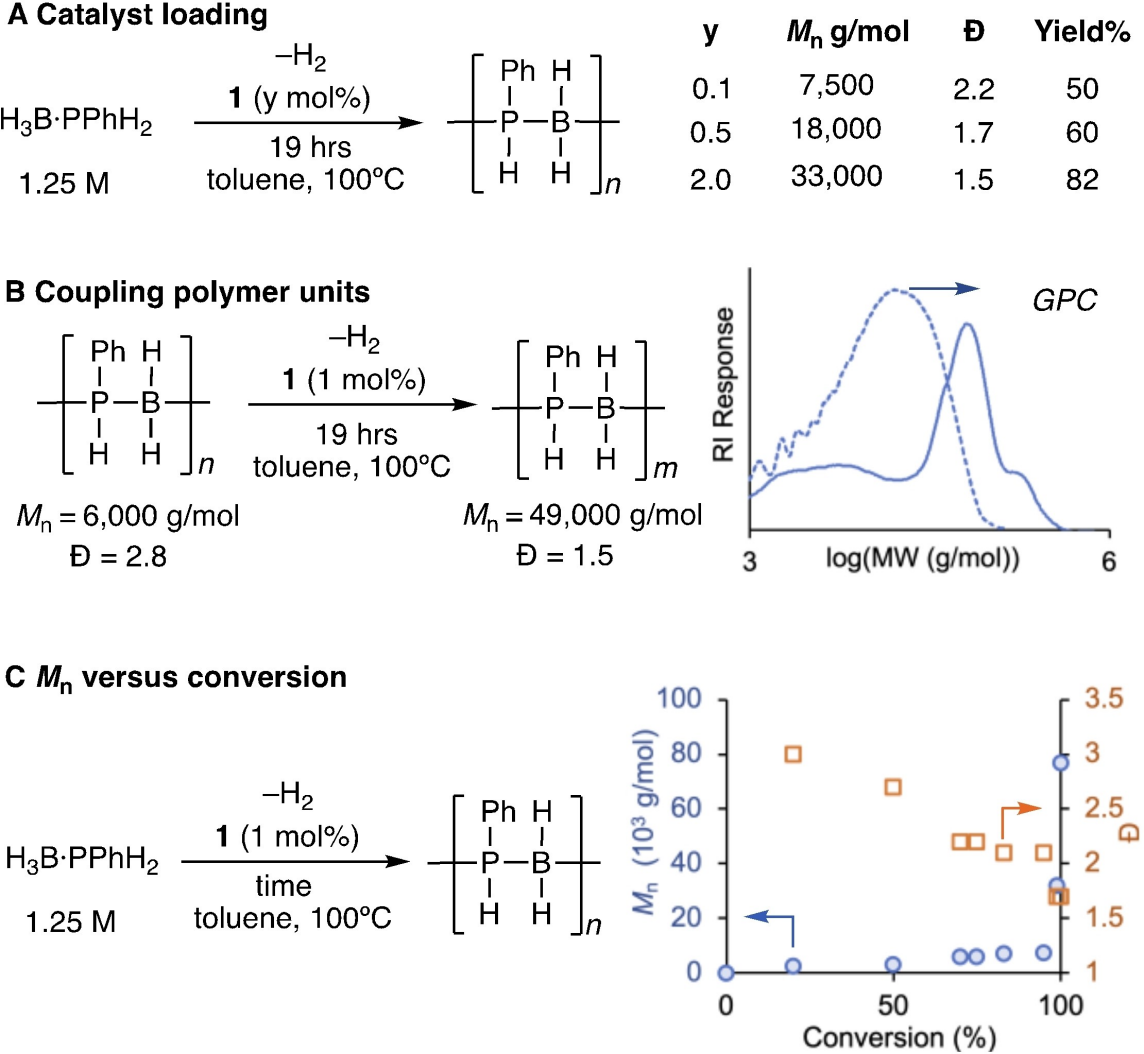
Experiments to probe step‐growth propagation A) Changes in catalyst loading, yield=isolated yield; B) Dehydrocoupling of shorter polymers, dotted line shows initial shorter polymer; C) Molecular weight and dispersity versus conversion. Conversion=conversion of H_3_B ⋅ PPhH_2_. 99 % conversion was reached after 19 hrs, the final point was taken after 5 days (*M*_n_=77 000 g mol^−1^).

To probe this step‐growth like mechanism further, a number of experiments were conducted. Firstly, variation in catalyst loading from 0.1 mol% to 2 mol% resulted in an increase in *M*_n_ of isolated polymer using higher loadings, but incomplete conversions with higher dispersities at lower loadings using the same reaction time (Scheme 4A). These observations are consistent with a step‐growth mechanism, where higher catalyst loadings promote more coupling events.^[35]^ This is in contrast to catalyst **II** where lower loadings resulted in higher *M*_n_, that was used as evidence for a different, chain‐growth, mechanism operating.^[33]^ Secondly, catalyst **1** is able to couple shorter polymers to form longer polymer chains, Scheme 4B. Thirdly, a conversion/*M*_n_/*Ð* plot (by quenching individual experiments at different times) clearly demonstrates step growth propagation (Scheme 4C), with high MW polymer only formed at very high conversions of monomer, that is also accompanied by a decrease in dispersity.[^35^, ^46^] Very high MW polymer (*M*_n_=77 000 g mol^−1^) is only formed after 5 days reaction time. In contrast, for a chain growth process, high molecular weight polymer would be expected to be observed at low conversions of monomer.[^33^, ^47^] Collectively these observations support a step‐growth mechanism where reversible chain transfer occurs, i.e. H_3_B ⋅ PPhH_2_/oligomer/polymer can all coordinate with, and be activated by, the catalyst. By harnessing this mechanism, we next show that novel block copolymers of polyphosphinoboranes can be synthesized.

It is well established that GPC/RI detection using conventional column calibration (CCC) against polystyrene standards overestimates the degree of polymerization in polyaminoboranes, by between a factor of 3 to 10, due to the fundamental chemical differences between group 13/15 and hydrocarbon‐based polymers (e.g., BH/NH versus CH).[^48^, ^49^] It is likely a similar relationship holds for polyphosphinoboranes. Thus, to characterize any block copolymers formed a more accurate representation of degree of polymerization is required, and this comes from multi‐angle light scattering (MALS) detection. Using a variety of polyphosphinoborane samples produced in the study here allows for a calibration graph that relates *M*_n_ data from MALS with those from CCC (Figure S35). This shows that, on our instrumentation set up (Supporting Materials), CCC overestimates *M*_n_ approximately 3–4‐fold for polymers measured between 80 000 and 20 000 g mol^−1^ using CCC, and likely even higher for lower molecular weight polymers/oligomers.

**Scheme 5:**
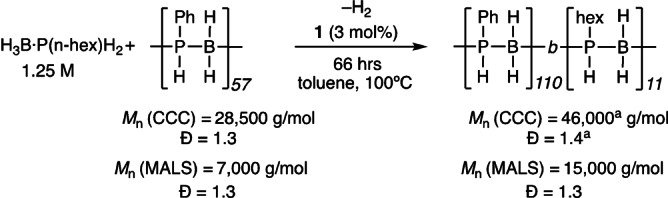
Synthesis of [H_2_BPPhH]_110_‐block‐[H_2_BP(n‐hexyl)H]_11_. CCC=Conventional Column Calibration. ^a^ Data from the major, high‐molecular weight component.

**Figure 1:**
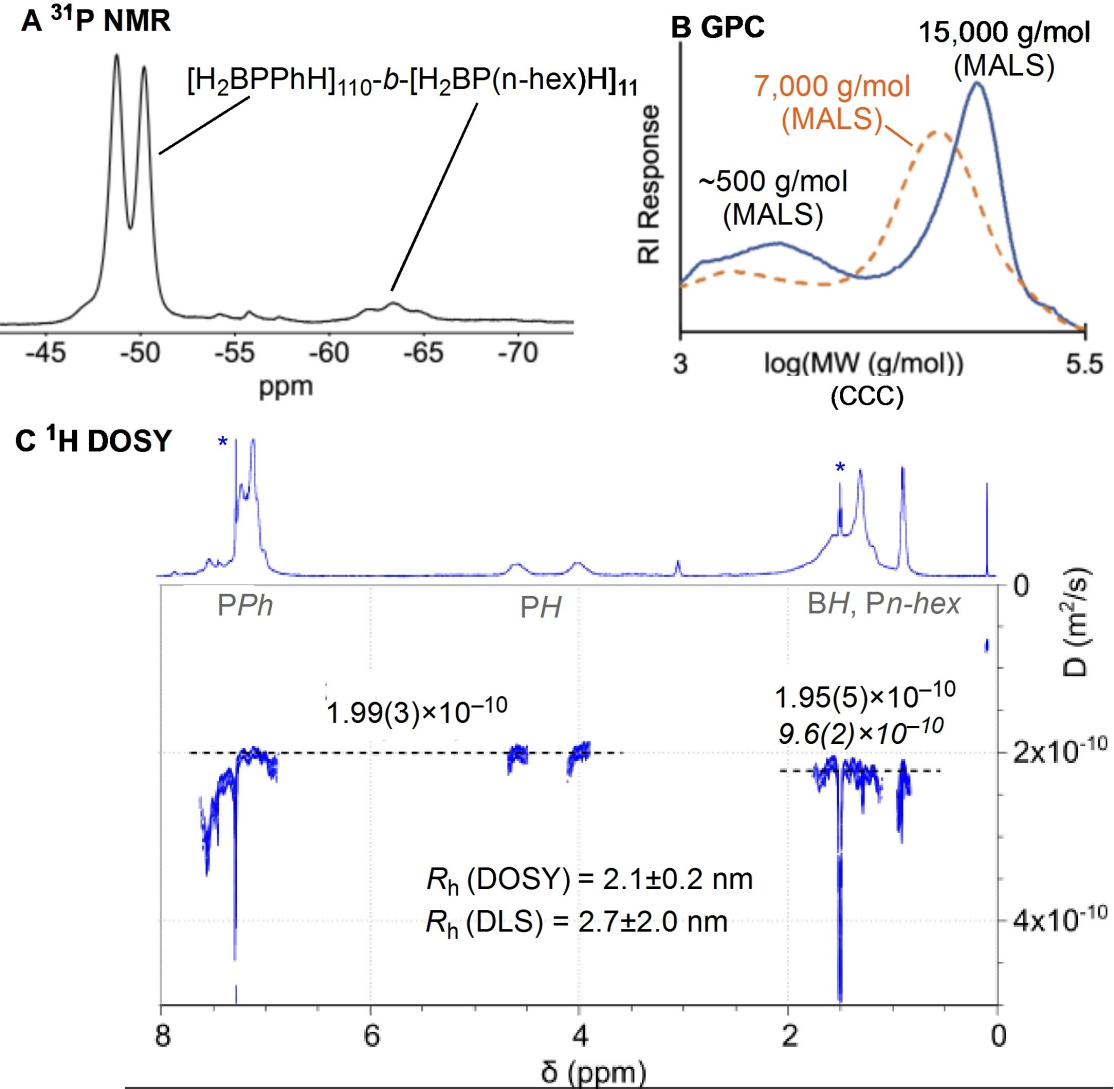
Solution characterization data for **BCP1**. A) ^31^P NMR spectrum (CDCl_3_); B) GPC (indicated *M*_n_ from MALS), dotted line represents initial homopolymer [H_2_BPPhH]_n_; C) ^1^H DOSY (CDCl_3_, 298 K, 600 MHz) using a 2‐term diffusion model for the aliphatic peaks (minor component in italics). R_h_=Apparent hydrodynamic radius from DOSY, R_DLS_=hydrodynamic radius from DLS. * residual solvent signals.

With this calibration in hand, taking pre‐formed [H_2_BPPhH]_n_, *M*_n_(MALS)=7 000 g mol^−1^, with H_3_B ⋅ P(n‐hexyl)H_2_ monomer (1.25 M in toluene), catalyst **1** (3 mol%), and heating for 66 hrs at 100 °C resulted in a mixture of products by ^31^P{^1^H} NMR spectroscopy, including homopolymer [H_2_BP(n‐hexyl)H]_n_ and related short‐chain oligomers. Using the noted solubility difference between Ph‐ and n‐hexyl substituted polyphosphinoboranes, extraction into hexanes selectivity removed polymers/oligomers containing only n‐hexyl groups. Importantly the hexane *insoluble* portion displayed both phenyl and hexyl resonances in the corresponding ^1^H and ^31^P spectra, an initial indication that a block copolymer had been formed, Scheme 5.^[50]^ For example in the ^31^P NMR spectrum a signal at δ −49.5 [*J*(PH)=350 Hz] is assigned to [H_2_BPPhH]_n_ segments, while a cluster of signals centered at −63.4 is assigned to [H_2_BP(n‐hexyl)H]_n_, interestingly in which the atacticity can now be observed,^[17]^ Figure 1A. The approximate ratio of these two distinctly different regions is 10 : 1. Interrogation of the ^1^H NMR spectrum in the P−H region indicates a similar ratio of Ph to n‐hexyl units. Additional smaller signals are observed in the ^31^P NMR spectrum at δ −55.8 as an apparent triplet, that simplifies to a singlet on decoupling ^1^H, and δ −48 (partially obscured). These could reflect connecting units between block segments, P(n‐hexyl)H_2_ end groups[^17^, ^34^] or hexane insoluble small oligomers. GPC analysis using MALS detection showed polymer with an increased degree of polymerization *M*_n_=15 000 g mol^−1^ (*Ð*=1.3; CCC, *M*_n_=46 000 g mol^−1^), Figure 1B. With the relative ratios from the NMR spectra and the absolute molecular weight from MALS this approximates the composition of this new block copolymer to [H_2_BPPhH]_110_‐*b*‐[H_2_BP(n‐hexyl)H]_11_, **BCP1**. A small amount of low molecular weight oligomer (≈500 g mol^−1^) is also observed in the GPC spectrum. As pre‐formed [H_2_BPPhH]_n_ contained no such species, we assign this to short oligomers of [H_2_BP(n‐hexyl)H]_n_.

^1^H Diffusion Ordered Spectroscopy (DOSY, CDCl_3_, 298 K) provides further evidence for the formation of a block copolymer, Figure 1C. DOSY has been used previously for the analysis of polyphosphinoborane homopolymers^[30]^ as well as hydrocarbon‐based block copolymers.^[51]^ Taking each homopolymer of similar MW (CCC, *M*_n_=44 000–33 000 g mol^−1^) shows that [H_2_BPPhH]_n_ and [H_2_BP(n‐hexyl)H]_n_ have very different diffusion coefficients (1.5(2) and 3.9(8) ×10^−10^ m^2^ s^−1^, respectively, Figure S42). However, **BCP1** also has a different diffusion coefficient, that is the same for both phenyl and hexyl regions of the ^1^H NMR spectrum (1.99(3) and 1.95(5) ×10^−10^ m^2^ s^−1^), indicating both segments are in the same macromolecule. A two‐component model is needed to best fit the data in the alkyl region, with a minor, faster diffusing, component required (9.6(2)×10^−10^ m^2^ s^−1^). This is fully consistent with the shorter, oligomeric, material observed by GPC. Dynamic Light Scattering (DLS, CHCl_3_) experiments place the apparent hydrodynamic radius of **BCP1** as 2.7±2.0 nm in CHCl_3_ (Fig S44), a reasonable fit with that from DOSY (2.1±0.2 nm), and supports the formation of non‐aggregated polymer chains in CHCl_3_ solution.

While we currently cannot comment on the precise order of events to form **BCP1**, the ability to reversibly bind and activate different terminus P−H/B−H groups is no doubt important to the formation of a block copolymer. As it is well established that P−H activation occurs more quickly with PPhH_2_ than with P(n‐hexyl)H_2_ (Table 1), and also that catalyst **1** will couple pre‐formed polymer (Scheme 4), we suggest that a plausible order of events is a relatively rapid coupling of shorter [H_2_BPPhH]_n_ units, with the more reactive ‐PPhH_2_ terminus (Scheme 3C), followed by slower sequential step‐growth addition H_3_B ⋅ P(n‐hexyl)H_2_ or equivalent short chain oligomers.

**Figure 2:**
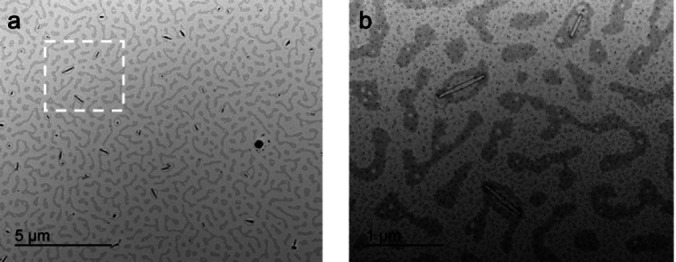
TEM images of assembled structures of [H_2_BPPhH]_110_‐b‐[H_2_BP(n‐hexyl)H]_11_, **BCP1**, obtained from a 37.5 %:62.5 % v/v THF:Hexane solution, without staining and after allowing the solvent to evaporate. The structures highlighted in the box in (a) are shown with a fivefold magnification in (b). The carbon film substrate also shows evidence for the deposition of a unimer film formed by residual molecularly dissolved **BCP1**. The very dark regions in the images are believed to arise from electron‐dense residual Rh catalyst‐derived impurities. Qualitative analysis for residual Rh‐content in **BCP1** using inductively coupled plasma mass spectrometry (ICP‐MS) revealed a significant amount is retained (ca. 1 ppm).

Amphiphilic block copolymers are well‐known to self‐assemble in solvents that are selective for one of the constituent segments to form core–shell nanoparticles or micelles.[^5^, ^52^] By utilizing the different solubilities of the blocks present in **BCP1** self‐assembled micellar nanostructures should be formed in solution. Molecularly dissolved BCP unimers were observed in pure THF and CHCl_3_, or in an equal volume mixture of THF and hexane by dynamic light scattering (DLS) (hydrodynamic radius R_h_<5 nm, Figure S44 and Table S6). When the volume fraction of hexane was increased to 37.5 %:62.5 % v/v THF:hexane, to enhance the selectivity for the −[H_2_BP(n‐hexyl)H]_11_ segment of **BCP1**, DLS provided evidence for the formation of self‐assembled aggregates with R_h_=132±12 nm (Figure S44). Decreasing the solvent polarity further to 25 %:75 % THF:hexane resulted in larger aggregates being formed (R_h_=180±30 nm, Figure S44 and Table S6). The solvent composition and DLS data indicate that micelles are formed with a core of the more polar −[H_2_BPPhH]_110_ block and a corona of the less polar −[H_2_BP(n‐hexyl)H]_11_ segment. The core:corona block ratio ca. 10 : 1 classifies these systems as crew‐cut micelles and non‐spherical morphologies, rather than star‐like micelles, would be anticipated based on packing‐parameter considerations.^[5]^ Indeed, TEM images of drop‐cast aliquots of **BCP1** in 37.5 %:62.5 % v/v THF:hexane solution revealed the formation of rod‐like micelles (10 nm×≈500 nm in dimensions, Figure 2). In the less polar solvent mix (25 %:75 % THF:hexane) assemblies with a spherical shape were formed which were even larger in dimensions (≈250 nm, Figure S45). These assemblies are far too large to be conventional star‐like micelles and are most likely either compound micelles or, more likely, vesicles.^[5]^

## Conclusion

The synthesis of block copolymers of polyphosphinoboranes, and their self‐assembly in solution to form well‐defined micellar structures, demonstrates that these main‐group polymers, that are valence isoelectronic with polyolefins, have the potential to have rich nanoscale properties. The step growth‐like mechanism that operates, using precatalysts such as **1**, suggests future opportunities for bespoke tuning of the macromolecular properties in these group 13/15 polymers by variation of monomer functional group.^[53]^ It will also be interesting to see if the methods described here can be extended to other catalyst systems that promote dehydropolymerization of phosphine–boranes.

## Conflict of interest

The authors declare no conflict of interest.

1

## Supporting information

As a service to our authors and readers, this journal provides supporting information supplied by the authors. Such materials are peer reviewed and may be re‐organized for online delivery, but are not copy‐edited or typeset. Technical support issues arising from supporting information (other than missing files) should be addressed to the authors.

Supporting Information

## Data Availability

The data that support the findings of this study are available in the supplementary material of this article.
