# Supplementary material for: Polyphosphinoborane Block Copolymer Synthesis Using Catalytic Reversible Chain‐Transfer Dehydropolymerization
Source: Angew Chem Int Ed Engl. 2022 Dec 8;62(3):e202216106. doi: 10.1002/anie.202216106 (PMC10107156; doi:10.1002/anie.202216106)
Supplement: Supplementary file 1 — Supporting Information [file ANIE-62-0-s001.pdf]

Angewandte Chemie
Eine Zeitschrift der Gesellschaft Deutscher Chemiker

Supporting Information

Polyphosphinoborane Block Copolymer Synthesis Using Catalytic Reversible Chain-Transfer Dehydropolymerization

J. J. Race, A. Heyam, M. A. Wiebe, J. Diego-Garcia Hernandez, C. E. Ellis, S. Lei, I. Manners, A. S. Weller**

Table of Contents

General experimental methods S2
General procedure for preparation of $[H_2BPArH]_n$ S3
General Procedure for Preparation of $[H_2BP(n\text{-}hex)H]_n$ S8
Anion testing and catalyst loading experiments S10
Screening of different phosphines S11
Molecular Weight vs Conversion experiments S13
In-situ ^1H NMR monitoring S14
  ^1H NMR S14
  Re-charge experiment S15
Linear dimer ($H_3B{\cdot}PPhHBH_2{\cdot}PPhH_2$) preparation and reactivity studies S17
Speciation and stoichiometric experiments S20
Mechanism support – dehydrocoupling of oligomeric $[H_2BPPhH]_n$ S25
MALS GPC S26
Formation of $[H_2BPPhH]_{110}$-*b*-$[H_2BP(n\text{-}hex)H]_{11}$ BCP1 .... S27
  GPC Analysis S30
  ^1H DOSY NMR experiments S31
  Dynamic Light Scattering S34
  Transmission electron microscopy S35

General experimental methods

All experiments were performed under an atmosphere of argon, using standard Schlenk techniques on a dual vacuum/inlet manifold unless specified. Glassware was dried in an oven at 140 °C overnight or flame dried under vacuum prior to use. Hexane, THF and toluene were dried using an MBraun SPS-800 solvent purification system and degassed by three freeze-pump-thaw cycles. 1,2-$F_2C_6H_4$ was stirred over Al_2O_3 for two hours and then CaH_2 overnight before vacuum transfer and subsequent degassing by three freeze-pump-thaw cycles. Toluene-D_8 was dried overnight with KOH before vacuum transfer and subsequent degassing by three freeze-pump-thaw cycles and was stored over 3 Å molecular sieves.

$[Rh(dppe)_2]Cl$ (dppe = 1,2-bis(diphenylphosphino)ethane) was prepared via addition of dppe to $[Rh(COD)Cl]_2$ in THF (THF = tetrahydrofuran and COD = cyclooctadiene). $[Rh(dppe)_2][X]$ (X = $[BF_4]^-$ and $[BAr^F{}_4]^-$ were prepared by the addition of NaX to $[Rh(dppe)_2]Cl$ in THF. $[Rh(COD)Cl]_2$,[1] $H_3B{\cdot}PPhH_2$[2] and $H_3B{\cdot}PH_2$3,5-$(CF_3)_2C_6H_3$[3] were prepared via literature procedures. $H_3B{\cdot}P$(n-hex)H_2 was prepared via the Grignard reagent, (n-hex)MgBr, a modification from the literature method.[4] All other reagents were purchased from commercial vendors and used as received.

NMR data was collected on a Bruker 500 MHz AVIIIHD or Bruker 600 MHz AVIIIHD wide bore. Residual protio solvent resonances were used as a reference for ^{1}H NMR spectra. ^{31}P NMR spectra were referenced externally to 85 % H_3PO_4. All chemical shifts (δ) are quoted in ppm and coupling constants in Hz.

Gel permeation chromatography (GPC) was performed on a Malvern Viscotek GPCmax chromatograph fitted with a refractive index (RI) detector. The triple + guard column configuration was contained within an oven (35 °C) and consisted of a porous styrene divinylbenzene copolymer with a maximum pore size of 1,500 Å. THF containing 0.1% w.w $[N^nBu_4]Br$ was used as the eluent at a flow rate of 1.0 ml min^{-1}. Samples were dissolved (2.0 mg ml^{-1}) in the eluent and filtered (0.2 μm pore size) before running. The calibration was conducted using a series of monodisperse polystyrene standards (M_n = 474 – 467,000 g mol^{-1}) obtained from Sigma-Aldrich. A multi-angle light scattering detector was also used for absolute molecular weight determination.

Air sensitive mass spectrometry using a bespoke N_2 filled glovebox[5] connected to a Bruker ESI-ion trap spectrometer was used for speciation experiments.

Dynamic light scattering (DLS) was performed using a Malvern Panalytical Zetasizer Pro instrument equipped with a laser with a wavelength of 633 nm and a detector oriented at 173° to the incident radiation. Samples of **BCP1** were prepared at a concentration of 0.1 mg/mL using solvents filtered through a 0.45 μm syringe filter, and added to an optical quartz glass cuvette. A minimum of five measurements were taken for each sample. The correlation function was acquired in real time and analysed with a function capable of modelling multiple exponentials (Cumulant analysis). This process enables the diffusion coefficients for the component particles to be extracted, and these were subsequently expressed as effective hydrodynamic radius (R_h) using the Stokes-Einstein relationship for coated nanospheres with core properties of polystyrene latex.

^{1}H DOSY NMR spectra were collected on a Bruker 600 MHz AVIIIHD spectrometer equipped with a room temperature 5mm BBO probe. The dstebpgp3s pulse sequence was used to compensate for convection within the sample. For all experiments, the number of scans per increment was 16, the delay between scans was 10 s, and the temperature was 298 K. The diffusion delay d20 and gradient pulse length p30 were optimised to give a roughly 95 % drop in polymer signal between the highest and lowest gradient strengths: this gave d20 = 200 ms and p30 = 1.2 ms. For **BCP1** 16 linearly spaced increments were collected between 5% and 95% gradient strength; for all other samples, only 8 linearly spaced increments were collected. The gradient strength was calibrated using a sample of 1% H_2O in D_2O, which is known to have a diffusion coefficient of 1.872 x 10^{-9} m^2 s^{-1}. No signs of eddy currents were observed. DOSY spectra were processed in using the dosy2d script in Bruker Topspin, with 1024 points in the indirect dimension. Bruker Dynamics Centre was used to fit the single or double diffusion models in Figure S43.

Transmission electron microscopy (TEM) was conducted using copper grids (400 mesh) purchased from Ted Pella, Inc. and carbon films were prepared by using a Leica EM ACE600 instrument. Carbon films were deposited onto the copper grids by flotation on water and allowed to dry over 24 hours. Samples for electron microscopy were prepared by drop-casting 8 µL of **BCP1** in a THF solution onto a carbon-coated copper grid followed by solvent evaporation. TEM images were obtained using a JEOL JEM 1011 operating at 80 kV, equipped with an 11 megapixel CCD camera.

General procedure for preparation of $[H_2BPArH]_n$

Toluene (0.2 ml) was added to a high pressure NMR tube containing $[Rh(dppe)_2]Cl$ (2.3 mg, 0.0025 mmol) and $H_3B{\cdot}PArH_2$ (0.25 mmol, 1.25 M) [Ar = Ph or 3,5-$(CF_3)_2C_6H_3$] and the NMR tube was sonicated for five minutes before being heated to 100 °C with aid of an oil bath and left at this temperature, without stirring, for the specified time, depending on the desired molecular weight. **Important – the NMR tube is kept sealed during the reaction and with 33 mg H_3BPPhH_2 (0.25 mmol) the pressure is 3.9 Bar at 100 °C from H_2 evolution**. **Be careful if scaling up**. The reaction mixture was removed from the oil bath and allowed to cool to room temperature. The mixture was then analysed by ^{31}P and ^{11}B NMR spectroscopy to determine the conversion of monomer. The solution was transferred by cannula to a Youngs Flask and then hexane was added with vigorous stirring, resulting in the formation of a white precipitate. The polymer was allowed to settle, and the solvent was removed by cannula and the white solid was dried under Schlenk line vacuum (< 1 x 10^{-1} mBar) for at least two hours before GPC analysis was conducted to determine the molecular weight of the polymer. Depending on the reaction time, different molecular weight polymer was formed, and the yield also varied, for example: after 19 hours polymer of M_n = 26,500 g mol^{-1} (Đ = 1.6) and 127,500 g mol^{-1} (Đ = 1.2) for Ar = Ph and 3,5-$(CF_3)_2C_6H_3$ respectively (see section on molecular vs conversion). The signals in the ^{31}P, ^{1}H and ^{11}B NMR spectra closely replicated the literature data.[3],[6]

$[H_2BPPhH]_n$

$^{31}P\{^1H\}$ (243 MHz, $CDCl_3$, 298 K): δ –49.5 (br s)

^{31}P (243 MHz, $CDCl_3$, 298 K): δ –49.5 (d, J_{PH} = 355 Hz)

^1H NMR (500 MHz, $CDCl_3$, 298 K): δ 7.19-6.75 (br m, 5H, P*Ph*H), 4.15 (br d, J_{PH} = 355 Hz, 1H,

PPh*H*), 1.37 (br s, 2H, BH_2).

^{11}B NMR (160 MHz, $CDCl_3$, 298 K): δ –33.8 (br s)

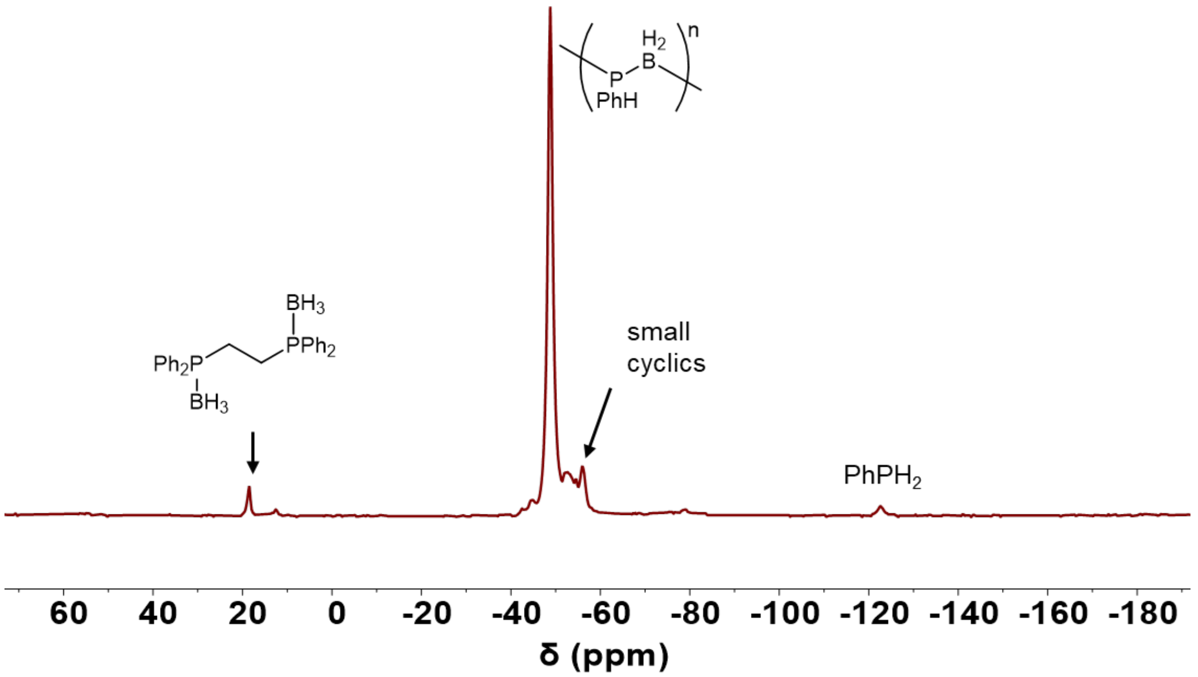

Figure S1. In-situ ^{31}P{^{1}H} NMR spectrum of $[H_2BPPhH]_n$ produced from the dehydropolymerisation of $H_3B{\cdot}PPhH_2$ using $[Rh(dppe)_2]Cl$ as a precatalyst, before polymer work up (1.25 M monomer in toluene, 1 mol% $[Rh(dppe)_2]Cl$, 100 °C, 19 hours) (toluene-D_8, 243 MHz, 298 K).

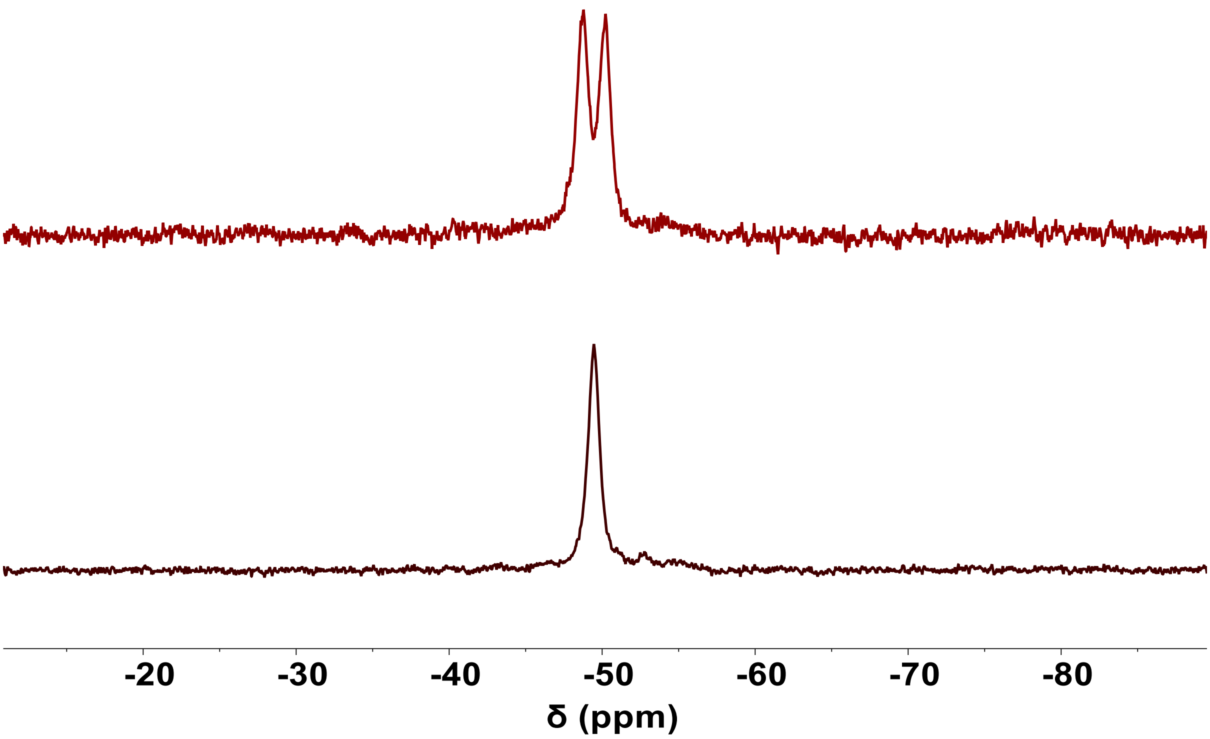

Figure S2. ^{31}P (top) and ^{31}P{^{1}H} (bottom) NMR spectra of $[H_2BPPhH]_n$ produced from the dehydropolymerisation of $H_3B{\cdot}PPhH_2$ using $[Rh(dppe)_2]Cl$ as a precatalyst (1.25 M monomer in toluene, 1 mol% cat., 100 °C, 19 hours) ($CDCl_3$, 243 MHz, 298 K).

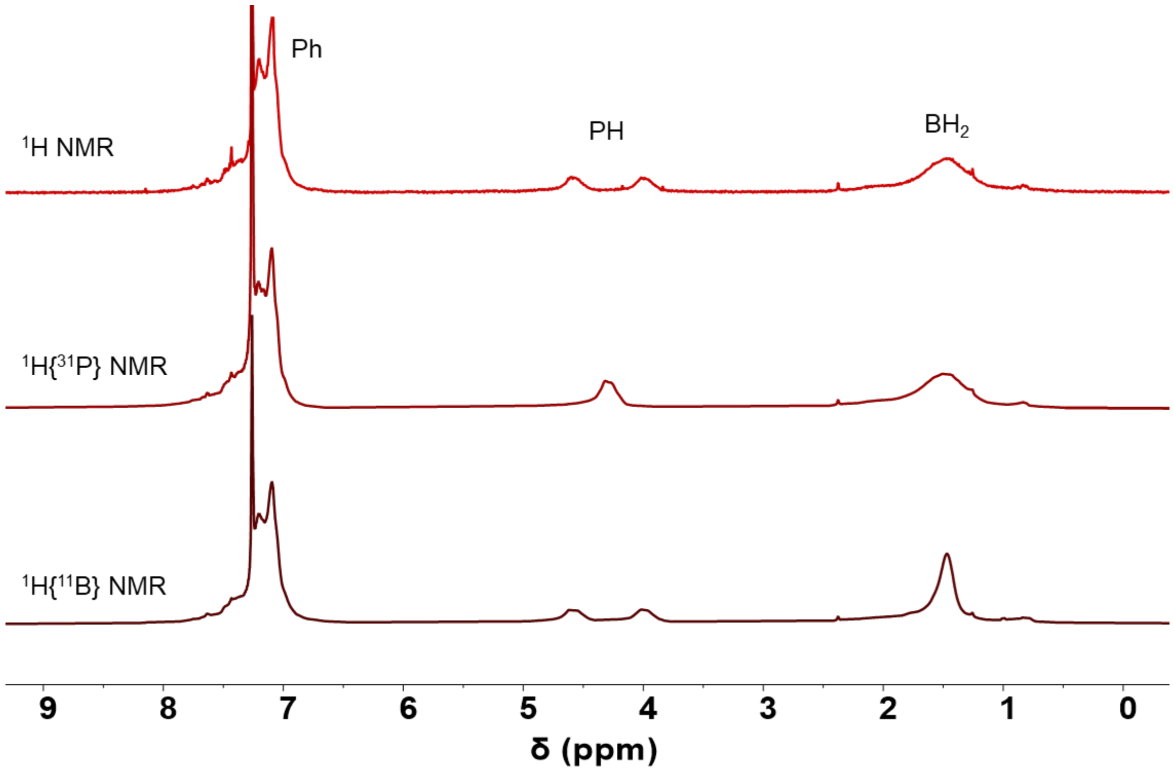

Figure S3. ^{1}H (top) and $^{1}H\{^{31}P\}$ (middle) and $^{1}H\{^{11}B\}$ (bottom) NMR spectra of $[H_2BPPhH]_n$ produced from the dehydropolymerisation of $H_3B{\cdot}PPhH_2$ using $[Rh(dppe)_2]Cl$ as a precatalyst (1.25 M monomer in toluene, 1 mol% $[Rh(dppe)_2]Cl$, 100 °C, 19 hours) ($CDCl_3$, 600 MHz, 298 K).

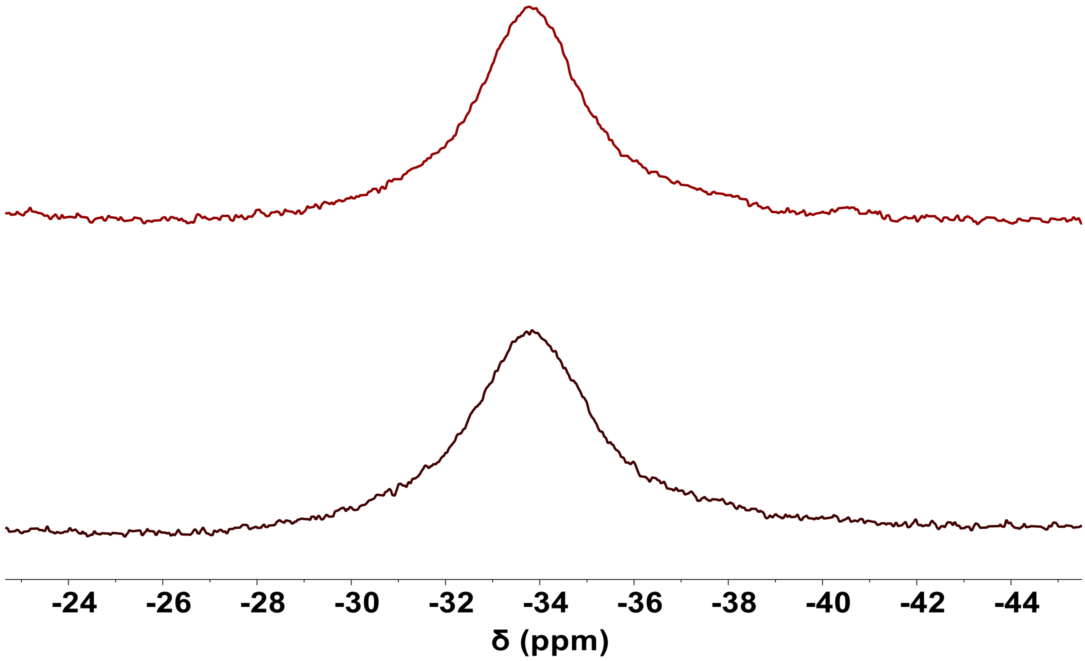

Figure S4. ^{11}B (top) and $^{11}B\{^{1}H\}$ (bottom) NMR spectra of $[H_2BPPhH]_n$ produced from the dehydropolymerisation of $H_3B{\cdot}PPhH_2$ using $[Rh(dppe)_2]Cl$ as a precatalyst (1.25 M monomer in toluene, 1 mol% $[Rh(dppe)_2]Cl$, 100 °C, 19 hours) ($CDCl_3$, 243 MHz, 298 K).

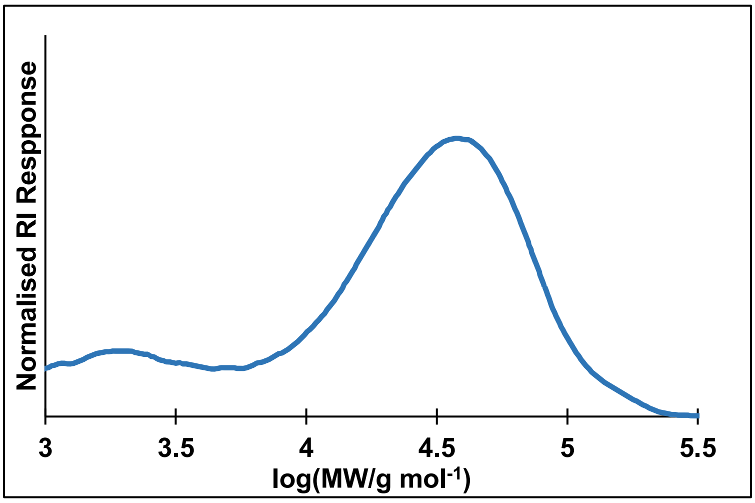

Figure S5. GPC trace of $[H_2BPPhH]_n$ produced from the dehydropolymerisation of $H_3B{\cdot}PPhH_2$ using $[Rh(dppe)_2]Cl$ as a precatalyst (1 mol%, toluene, 1.25 M, 100 °C, 19 hours). M_n = 26,500 g mol^{-1} and Đ = 1.5.

$[H_2BPArH]_n$ [Ar = 3,5-$(CF_3)_2C_6H_3$]

$^{31}P\{^1H\}$ (243 MHz, $CDCl_3$, 298 K): δ –45.6 (br s,), –46.9 (br s) and –48.3 (br s) in a 1:2:1 ratio.

^{31}P (243 MHz, $CDCl_3$, 298 K): multiplet with four broad peaks at: δ –44.8, –46.2, –47.7 and –49.1.

^1H NMR (500 MHz, $CDCl_3$, 298 K): δ 7.79-7.34 (br m, 5H, P*Ar*H), 4.60 (br d, J_{PH} = 361 Hz, 1H, PAr*H*), 1.57 (br s, 2H, BH_2).

^{11}B NMR (160 MHz, $CDCl_3$, 298 K): δ –35.0 (br s).

^{19}F NMR (376 MHz, $CDCl_3$, 298 K): δ –63.5 (br s).

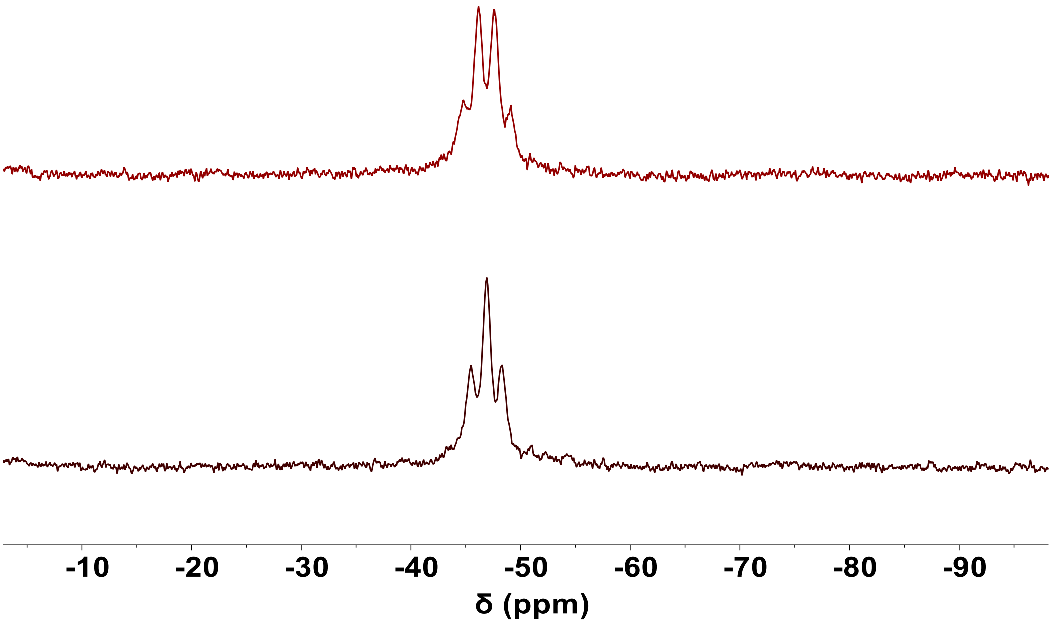

Figure S6. ^{31}P (top) and $^{31}P\{^1H\}$ (bottom) NMR spectra of $[H_2BPArH]_n$ [Ar = 3,5-$(CF_3)_2C_6H_3$] produced from the dehydropolymerisation of $H_3B{\cdot}PArH_2$ using $[Rh(dppe)_2]Cl$ as a precatalyst (1.25 M monomer in toluene, 1 mol% $[Rh(dppe)_2]Cl$, 100 °C, 19 hours) ($CDCl_3$, 243 MHz, 298 K).

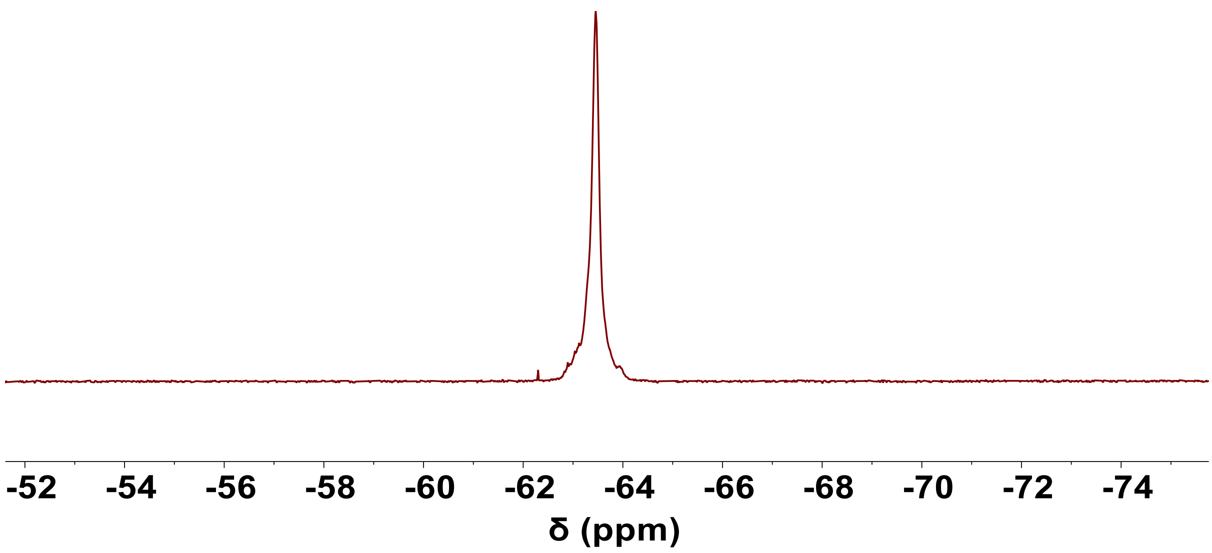

Figure S7. ^{19}F NMR spectrum of $[H_2BPArH]_n$ [Ar = 3,5-$(CF_3)_2C_6H_3$] produced from the dehydropolymerisation of $H_3B{\cdot}PArH_2$ using $[Rh(dppe)_2]Cl$ as a precatalyst (1.25 M monomer in toluene, 1 mol% $[Rh(dppe)_2]Cl$, 100 °C, 19 hours) ($CDCl_3$, 376 MHz, 298 K).

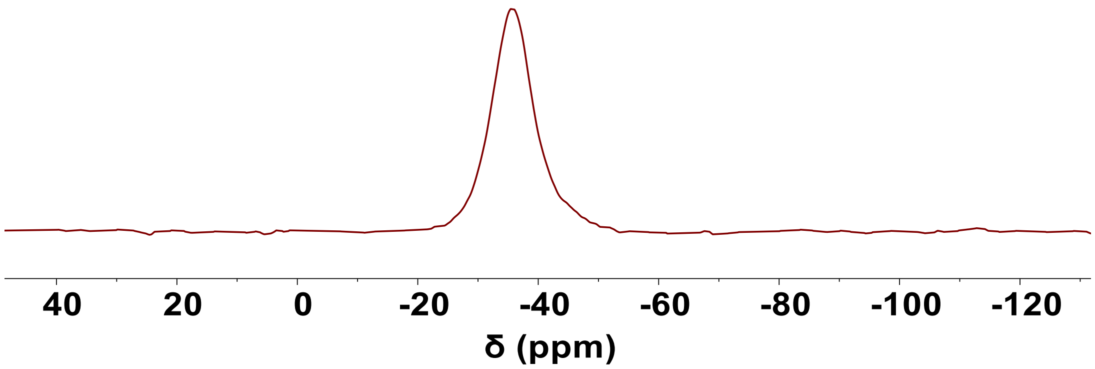

Figure S8. ^{11}B NMR spectrum of $[H_2BPArH]_n$ [Ar = 3,5-$(CF_3)_2C_6H_3$] produced from the dehydropolymerisation of $H_3B{\cdot}PArH_2$ using $[Rh(dppe)_2]Cl$ as a precatalyst (1.25 M monomer in toluene, 1 mol% $[Rh(dppe)_2]Cl$, 100 °C, 19 hours) ($CDCl_3$, 128 MHz, 298 K).

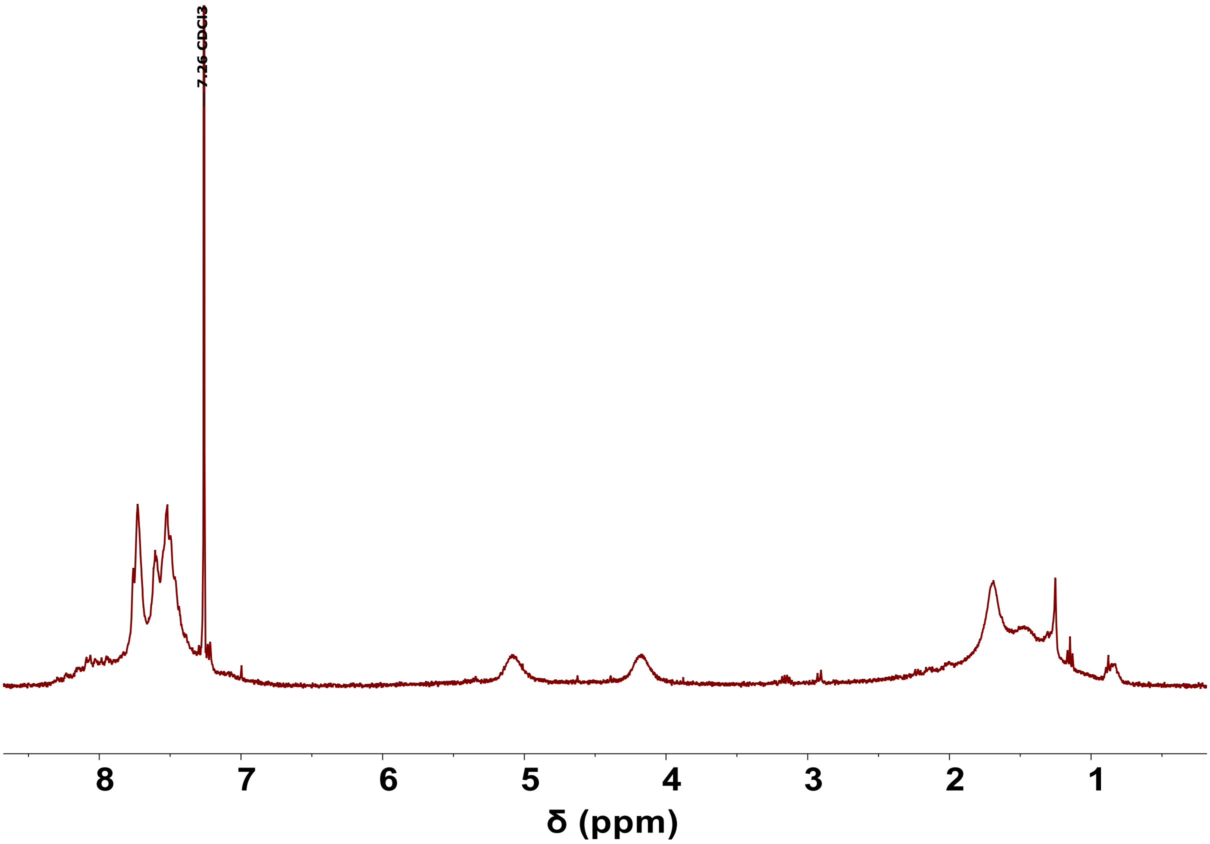

Figure S9. ^{1}H NMR spectrum of $[H_2BPArH]_n$ [Ar = 3,5-$(CF_3)_2C_6H_3$] produced from the dehydropolymerisation of $H_3B{\cdot}PArH_2$ using $[Rh(dppe)_2]Cl$ as a precatalyst (1.25 M monomer in toluene, 1 mol% $[Rh(dppe)_2]Cl$, 100 °C, 19 hours) ($CDCl_3$, 400 MHz, 298 K).

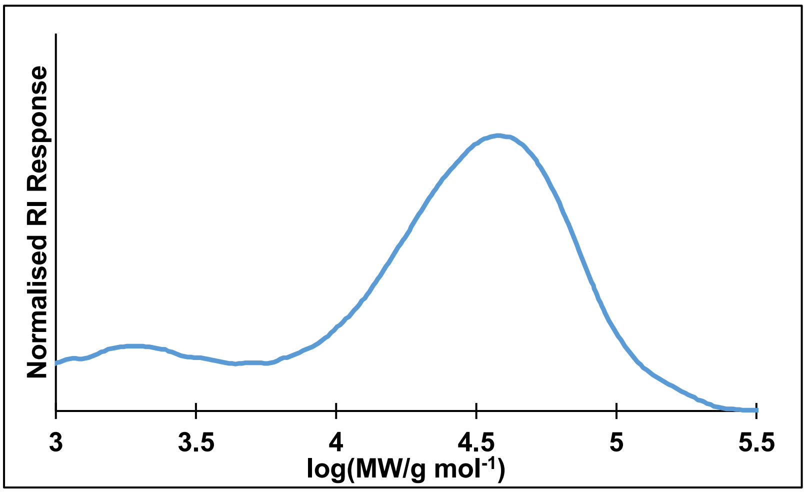

Figure S10. GPC trace of polymeric $[H_2BPArH]_n$ [Ar = 3,5-$(CF_3)_2C_6H_3$] produced from the dehydropolymerisation of $H_3B{\cdot}PArH_2$ using $[Rh(dppe)_2]Cl$ as a precatalyst (1 mol% $[Rh(dppe)_2]Cl$, toluene, 1.25 M, 100 °C, 19 hours). M_n = 127,500 g mol^{-1} and Đ = 1.2 by CCC.

General Procedure for Preparation of $[H_2BP(n\text{-}hex)H]_n$

Toluene (0.2 ml) was added to a high pressure NMR tube containing $[Rh(dppe)_2]Cl$ (6.9 mg, 0.0075 mmol, 3 mol%) and $H_3B{\cdot}P(n\text{-}hex)H_2$ (33 mg, 0.25 mmol). The NMR tube was sonicated for five minutes

before being heated to 100 °C with aid of an oil bath and left at this temperature, without stirring, for 66 hours in the sealed NMR tube. The reaction mixture was removed from the oil bath and allowed to cool to room temperature. The solution was filtered through separate Florosil® and Celite® plugs using dichloromethane as the eluent. The solvent mixture was evaporated to dryness and the resultant colourless oil was dried under Schlenk line vacuum (< 1 x 10^{-1} mBar) for one hour before the polymer was purified with Florosil® and Celite® plugs using CH_2Cl_2 as the eluent. GPC analysis revealed the formation of polymer with M_n of 33,000 g mol^{-1} and Đ = 1.3. Signals in the ^{31}P, ^{1}H and ^{11}B NMR spectra replicated those reported in the literature.[7] In the ^{11}B NMR spectrum, a signal at δ –39 is also observed, this could coincide with the smaller molecular weight polymer observed in the GPC trace, which could be smaller cyclic species.[8]

$[H_2BP(n\text{-}hex)H]_n$

$^{31}P\{^{1}H\}$ (243 MHz, $CDCl_3$, 298 K): δ –63.5 (br s), –46.9 (br s) and –48.3 (br s).

^{31}P (243 MHz, $CDCl_3$, 298 K): δ –63.0 (br s).

^{1}H NMR (500 MHz, $CDCl_3$, 298 K): δ 3.75 (br d, J_{PH} = 345 Hz, 1H, P(n-hex)*H*), 1.68 (br m, 12H, BH_2 and $(CH_2)_5$) and 0.87 (br s, 3H, CH_3).

^{11}B NMR (160 MHz, $CDCl_3$, 298 K): δ –34.8 (br m).

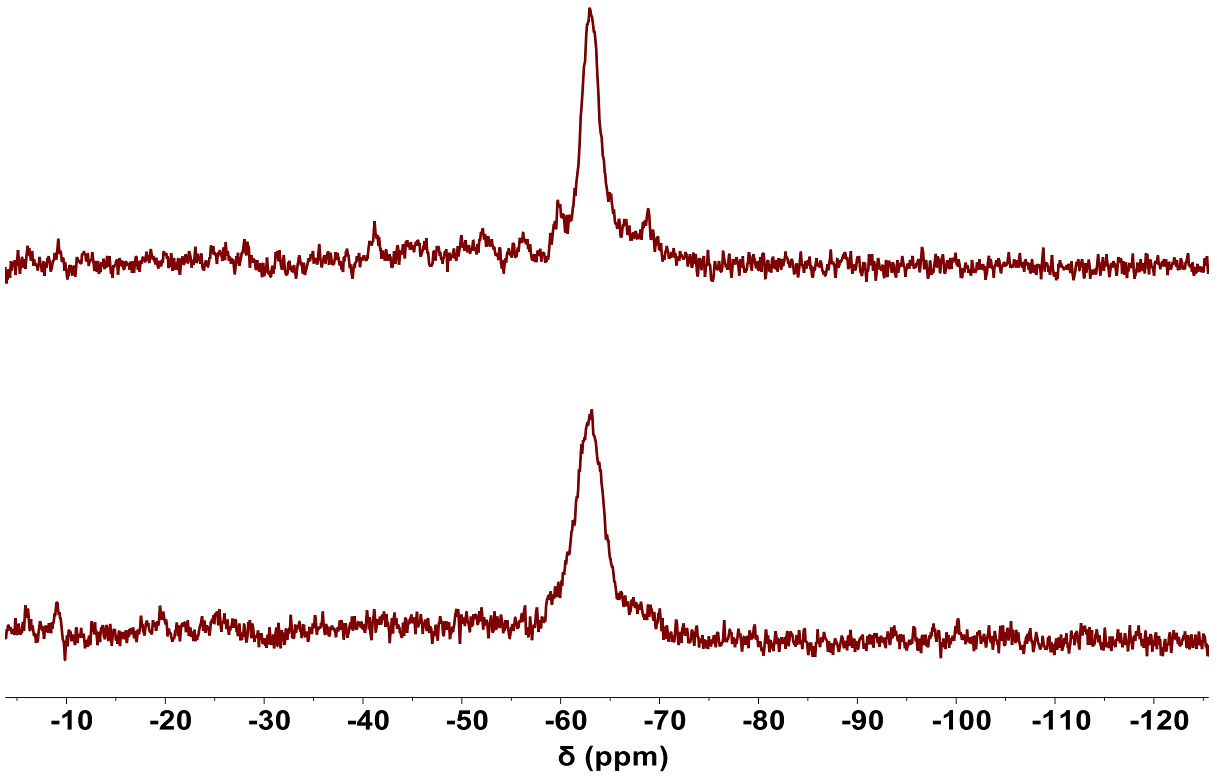

Figure S11. $^{31}P\{^{1}H\}$ (top) and ^{31}P (bottom) NMR spectra of poly(n-hexylphosphinoborane) from the dehydropolymerisation of $H_3B{\cdot}P(n\text{-}hex)H_2$ using $[Rh(dppe)_2]Cl$ as a precatalyst (reaction conditions: 1 mol% $[Rh(dppe)_2]Cl$, 1.25 M monomer, toluene, 100 °C, 66 hours) (243 MHz, toluene or $CDCl_3$, 298 K).

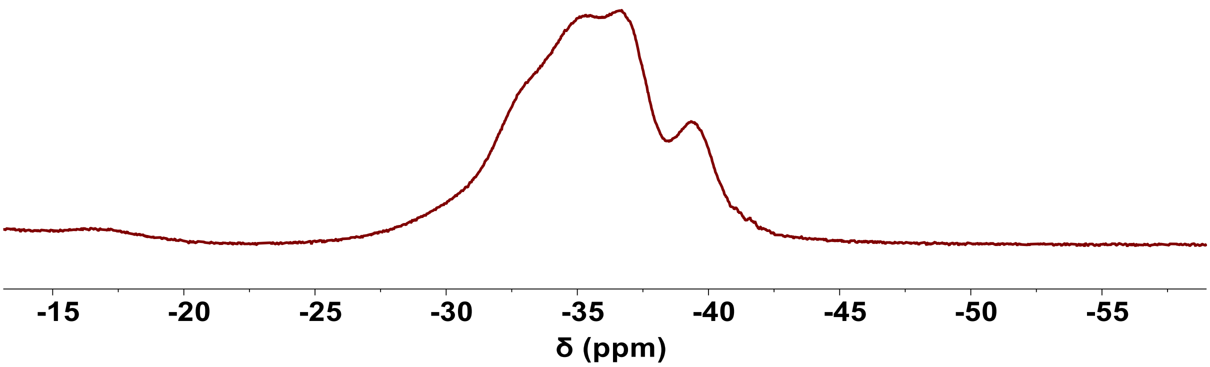

Figure S12. ^{11}B NMR of the post work-up mixture of products from the dehydropolymerisation of $H_3B{\cdot}P(n\text{-hex})H_2$ using the precatalyst [Rh(dppe)$_2$]Cl (reaction conditions: 3 mol%, 1.25 M, toluene, 100 °C, 66 hours) (193 MHz, $CDCl_3$, 298 K).

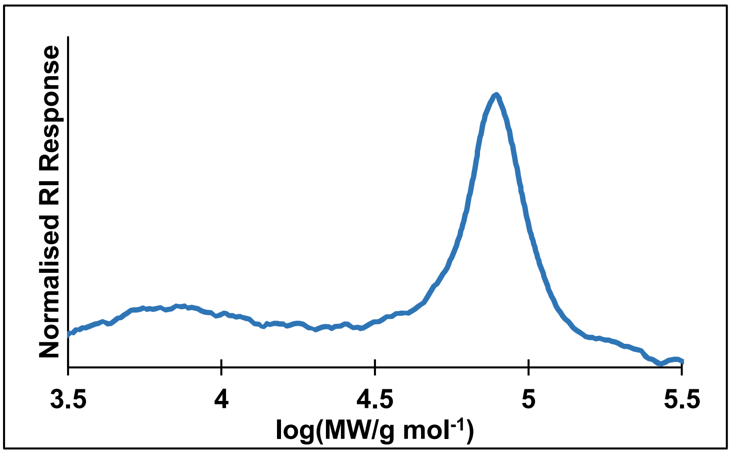

Figure S13. GPC trace of poly(n-hexylphosphinoborane) produced from the dehydropolymerisation of $H_3B{\cdot}P(n\text{-hex})H_2$ using [Rh(dppe)$_2$]Cl as a precatalyst (3 mol% [Rh(dppe)$_2$]Cl, toluene, 1.25 M, 100 °C, 66 hours). M_n = 33,000 g mol^{-1} and Đ = 1.3.

Anion testing and catalyst loading experiments

At 1 M $H_3B{\cdot}PPhH_2$ some lower molecular weight polymer was also formed, potentially oligomeric material, therefore proceeding experiments were conducted at 1.25 M (see figure below).

Table S1. Different precatalysts tested in the dehydropolymerisation of $H_3B{\cdot}PPhH_2$ (toluene, 100 °C, 19 hours). Molecular weight dispersity calculated by GPC analysis with conventional column calibration.

Catalyst	Cat. loading (mol%)	Starting monomer concentration (M)	M_n (g mol^{-1})[e]	PDI Đ	Yield[d] (%)
[Rh(dppe)$_2$][BAr$^F_4$]	1	1.0	13,500[a]	1.8	74
[Rh(dppe)$_2$][BF$_4$]	1	1.0	38,500	1.4	78
[Rh(dppe)$_2$]Cl	1	1.0	36,000	1.4	85
[Rh(dppe)$_2$]Cl	1	1.25	26,500	1.6	86
[Rh(dppe)$_2$]Cl	0.1	1.25	7,500[b]	2.2	51
[Rh(dppe)$_2$]Cl	0.5	1.25	18,000[c]	1.7	60
[Rh(dppe)$_2$]Cl	2.0	1.25	33,000	1.5	82

[a] The BAr$^F_4$ anion is observable in the RI trace of the GPC. This disturbs the molecular weight determination to give a very different Mn value. [b] Only 75% conversion of $H_3B{\cdot}PPhH_2$ obtained. [c] Only 90% conversion of $H_3B{\cdot}PPhH_2$ obtained. [d] Yield of hexane insoluble polymer including the low molecular weight component. [e] M_n not including low molecular portion.

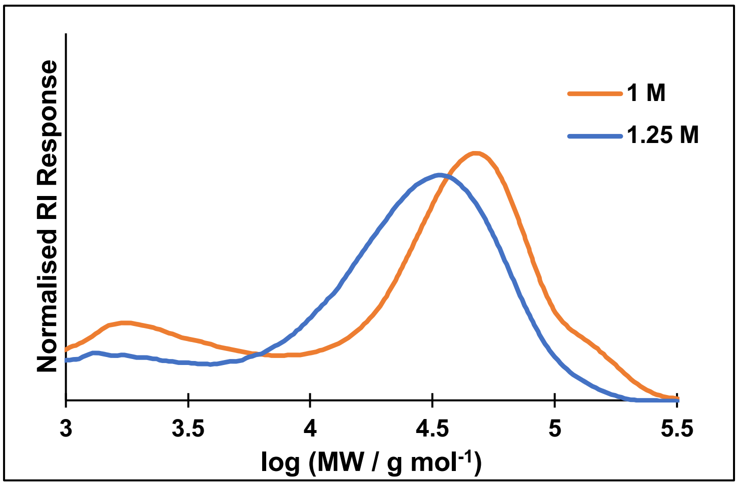

Figure S14. GPC trace of poly(phenylphosphinoborane) produced from the dehydropolymerisation of $H_3B{\cdot}PPhH_2$ using [Rh(dppe)$_2$]Cl as a precatalyst at 1 M and 1.25 M (1 mol%, toluene, 100 °C, 19 hours).

Screening of different phosphines

Instead of the general procedure in which [Rh(dppe)$_2$]Cl is added directly to the NMR tube, in these experiments, [Rh(COD)Cl]$_2$ (COD = cyclooctadiene) (0.6 mg, 1.25 μM) and four equivalents of phosphine (6 μM) were added to a high pressure NMR tube (see Table S2 for the phosphines used). $H_3B{\cdot}PPhH_2$ (31 mg, 0.25 mmol) then toluene (0.2 ml, 1.25 M) were also added to the NMR tube. The mixture was sonicated for 10 minutes before being heated to 100 °C in an oil bath and left at this temperature for 19 hours. The NMR tube was removed from the oil bath and the mixture was inspected by ^{31}P and ^{11}B NMR spectroscopy for completion of the reaction. The polymer mixture was transferred by cannula to a Youngs Flask and then hexane was added with vigorous stirring, resulting in the formation of a white precipitate. The polymer was allowed to settle, and the solvent was removed by cannula and the resultant white solid was dried under Schlenk line vacuum (< 1 x 10^{-1} mBar) for at least two hours before GPC analysis to determine the molecular weight of the polymer. In all cases, in the in-situ ^{31}P NMR spectra, complete conversion of the monomer was observed as well as the BH_3 adduct of free diphosphine. When only two equivalents of dppe was used, a bimodal distribution was observed, but when four equivalents was used a monomodal distribution was obtained.

Table S2. Different phosphines tested in the dehydropolymerisation of $H_3B{\cdot}PPhH_2$ (1.25 M monomer, 0.5 mol% $[Rh(COD)Cl]_2$, 2 mol% phosphine, toluene, 100 °C for 19 hours). * Two equivalents of dppe used.

**Ligand**	**M_n (g mol^{-1})**	**PDI Đ**	Modality
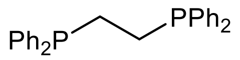	58,000*	1.8*	Bimodal*
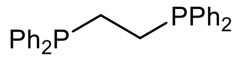	44,500	1.5	Monomodal
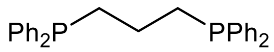	29,500	1.7	Monomodal
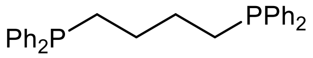	32,500	1.7	Monomodal
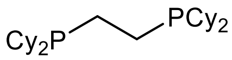	41,000	1.8	Monomodal
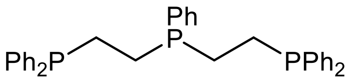	28,000	1.9	Monomodal

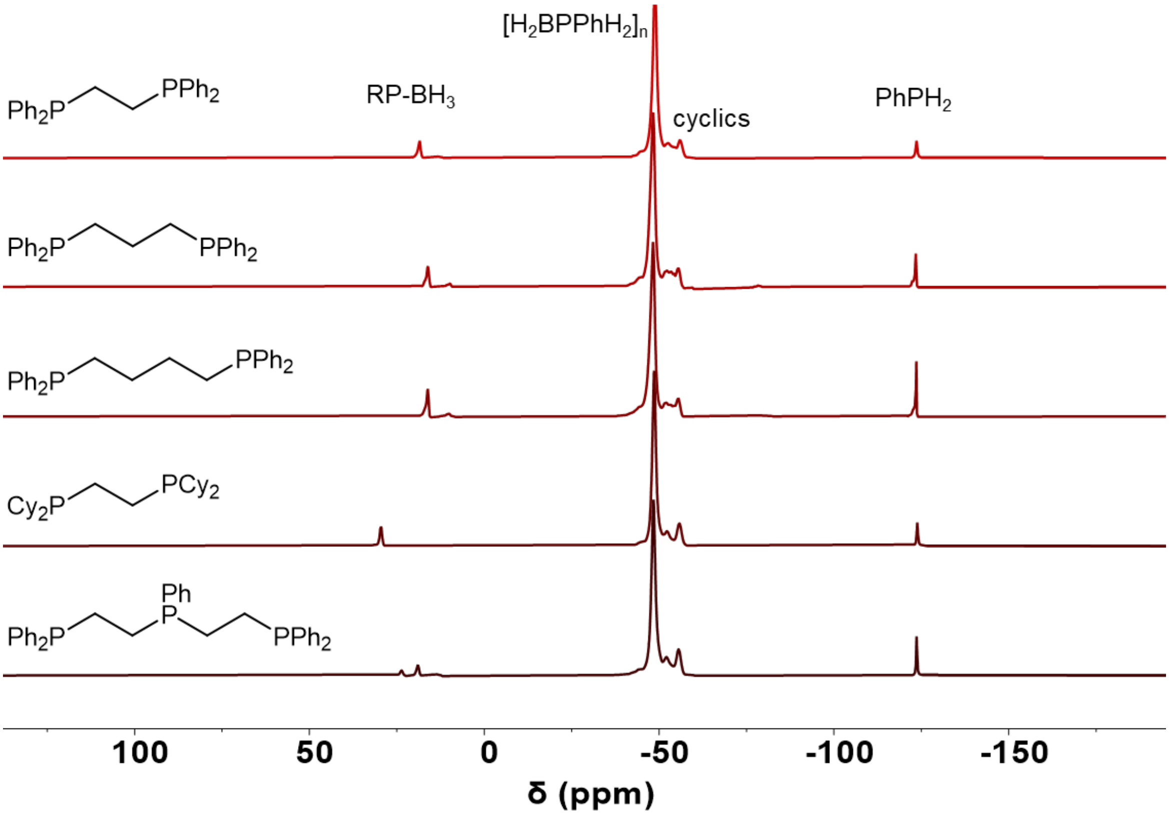

Figure S15. In-situ $^{31}P\{^1H\}$ NMR of the catalytic reaction mixture with different phosphines (1.25 M $H_3B{\cdot}PPhH_2$, 0.5 mol% $[Rh(COD)Cl]_2$, 2 mol% phosphine, toluene, 100 °C for 19 hours) (202 MHz, toluene-H_8, 298 K).

Molecular Weight vs Conversion experiments

The general procedure was used to synthesise and isolate poly(phenylphosphinoborane) of different molecular weight by using different reaction times at 100 °C in toluene. The molecular weight of the polymer was determined by GPC and plotted against the conversion of monomer, which was determined by in-situ ^{11}B NMR spectroscopy.

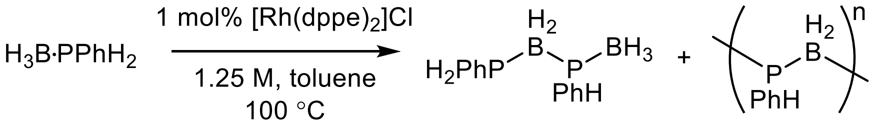

Scheme S1. Formation of $[H_2BPPhH]_n$ and $Ph_2PH·BH_2PPhH·BH_3$ from the dehydrocoupling and dehydropolymerisation of $H_3B·PPhH_2$.

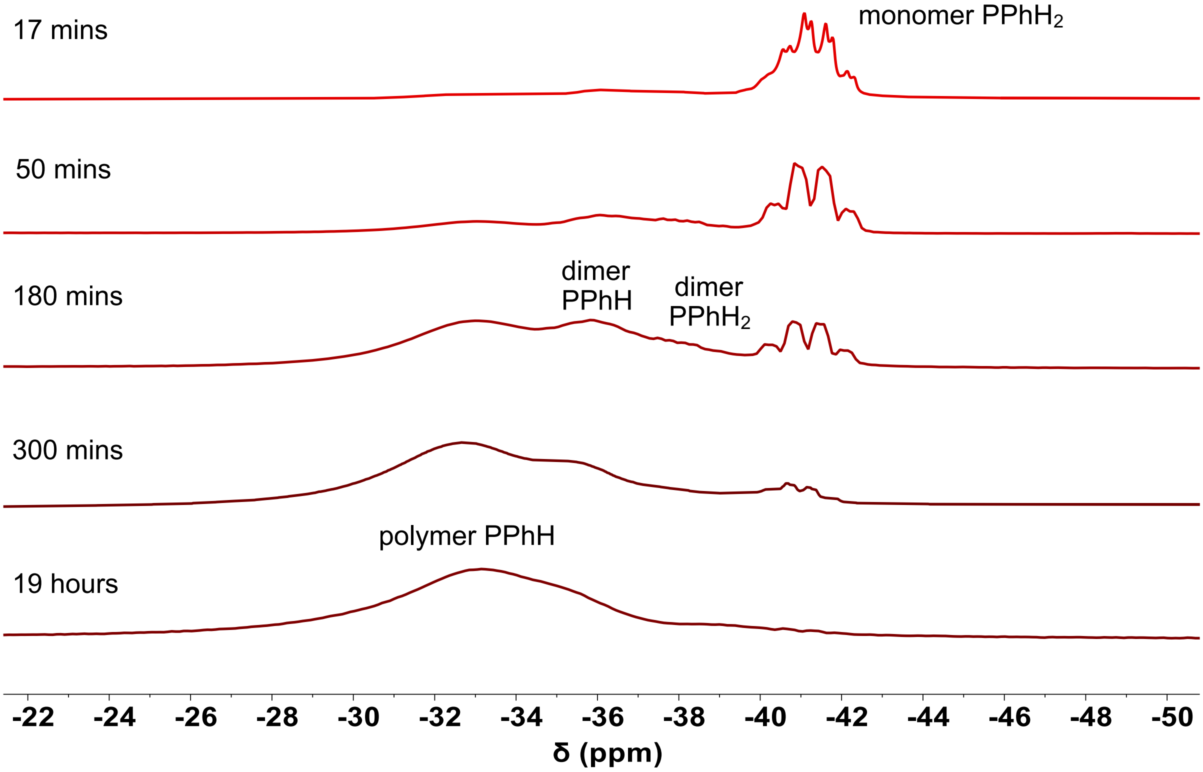

Figure S16. ^{11}B NMR spectra of the catalytic mixture of $H_3B·PPhH_2$ with 1 mol% $[Rh(dppe)_2]Cl$ in toluene (1.25 M) stopped after different periods of time at 100 °C (193 MHz, toluene, 298 K).

Table S3. Conversion, yield, polymer molecular weight and PDI from the dehydropolymerisation of $H_3B{\cdot}PPhH_2$ using $[Rh(dppe)_2]Cl$ as a precatalyst after different periods of time (1.25 M monomer, toluene, 100 °C). Molecular weight and dispersity calculated by GPC analysis with an RI detector.

Time (minutes)	Conversion[a] (%)	M_n (g mol^{-1})	PDI Đ	Yield[b] (%)
17	20	2500	3	15
50	50	3000	2.7	38
90	70	6000	2.2	56
130	75	6000	2.2	58
180	83	7000	2.1	60
300	95	7500	2.1	74
1140	99	3200	1.7	79
7200	100	77000	1.7	85

[a] Conversion of $H_3B{\cdot}PPhH_2$ as determined by ^{11}B NMR spectroscopy. [b] Yield of isolated polymer including any lower molecular weight component.

In-situ ^{1}H NMR monitoring

^{1}H NMR

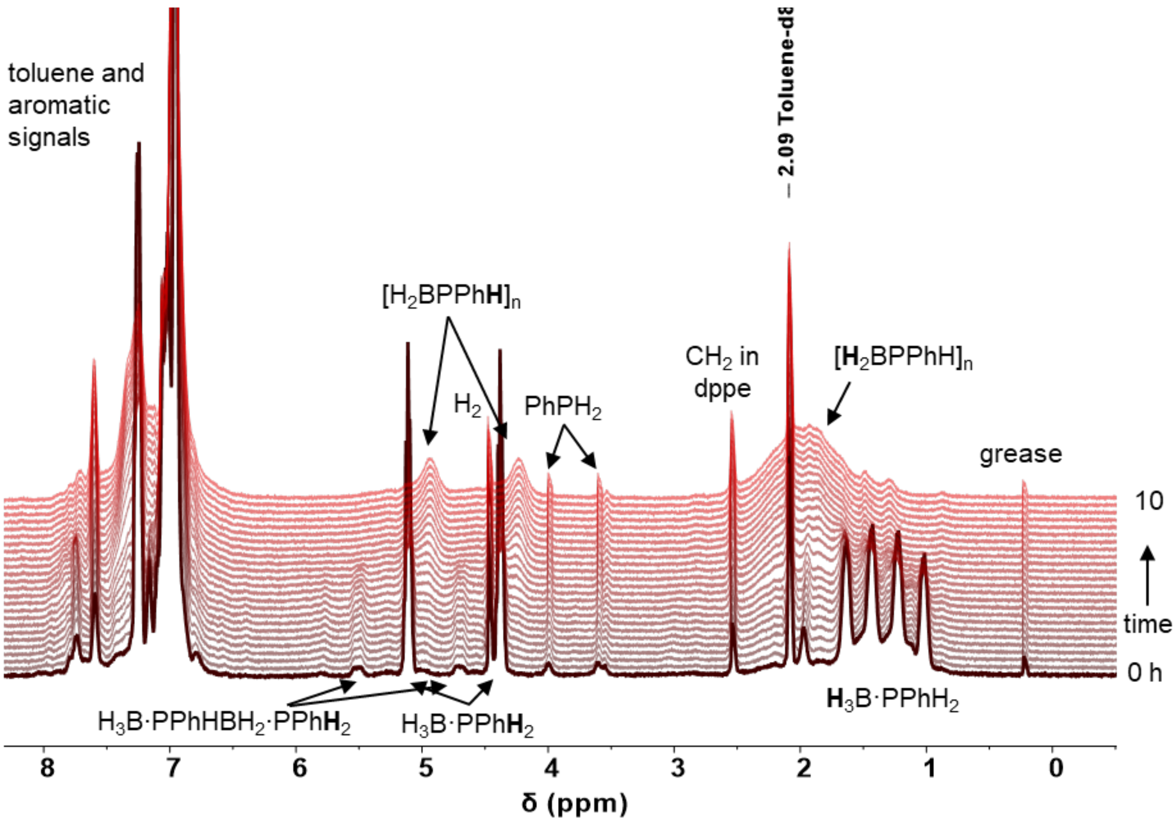

Figure S17. In-situ ^{1}H NMR of the dehydropolymerisation of $H_3B{\cdot}PPhH_2$, catalytic conditions: 3 mol% $[Rh(dppe)_2]Cl$, 0.25 M monomer in toluene-D_8, 373 K (500 MHz). Darker lines are earlier in the experiment and lighter red lines are at the end of the 10-hour experiment.

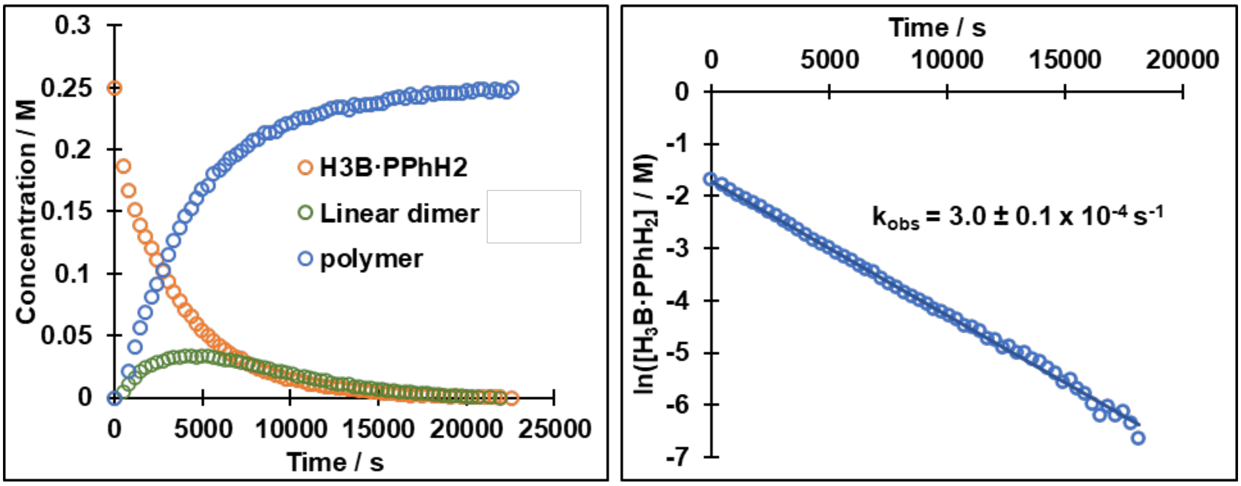

Figure S18. Left- relative integral plots of $H_3B{\cdot}PPhH_2$, $[H_2BPPhH]_n$ and linear dimer over time during the dehydropolymerisation of $H_3B{\cdot}PPhH_2$ using **4.1** as a precatalyst. Integrals relative to the initial concentration of $H_3B{\cdot}PPhH_2$. Right- the first order integrated rate plot of $[H_3B{\cdot}PPhH_2]$.

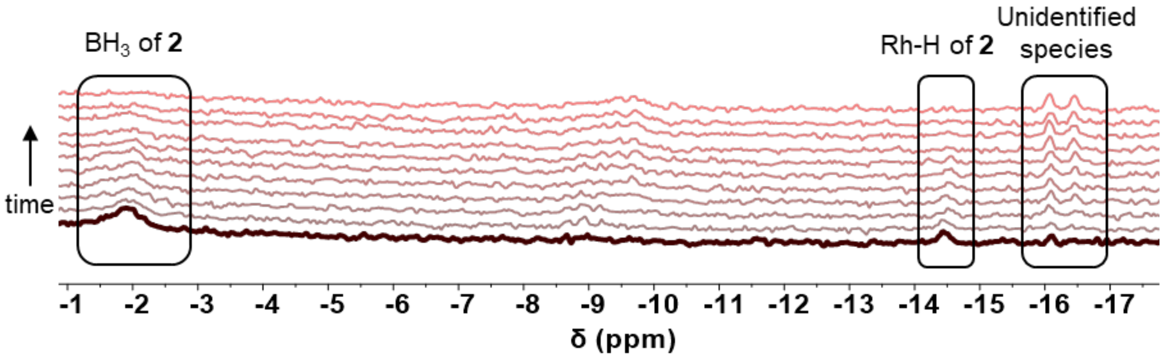

Figure S19. Hydride region of the in-situ ^{1}H NMR of the dehydropolymerisation of $H_3B{\cdot}PPhH_2$, catalytic conditions: 3 mol% $[Rh(dppe)_2Cl]$, 0.25 M monomer in toluene-D_8, 373 K (500 MHz). Darker lines are earlier in the experiment and lighter red lines are at the end of the 10-hour experiment.

Re-charge experiment

$H_3B{\cdot}PPhH_2$ (16 mg, 0.13 mmol), $[Rh(dppe)_2]Cl$ (3.6 mg, 0.004 mmol) and toluene (0.5 ml, 0.25 M) were added to a high pressure NMR tube and then sonicated for five minutes. The NMR tube was then placed in a Bruker 500 MHz AVIIIHD NMR spectrometer set at 100 °C and the ^{1}H NMR was monitored over 4.5 hours. The NMR tube was then removed from the spectrometer and the reaction mixture was transferred via cannula to another NMR tube containing $H_3B{\cdot}PPhH_2$ (16 mg, 0.13 mmol) before the new reaction mixture was placed in the NMR spectrometer and left at 100 °C for a further 16 hours and monitored by in-situ ^{1}H NMR spectroscopy. The final polymer was isolated by the general method and the NMR spectra matched well with the formation of $[H_2BPPhH]_n$ and the molecular weight was determined by GPC analysis to be M_n = 33,000 g mol^{-1} with a polydispersity of 1.5. It is evident that the rate is slower after the re-charge, although it does not stop completely.

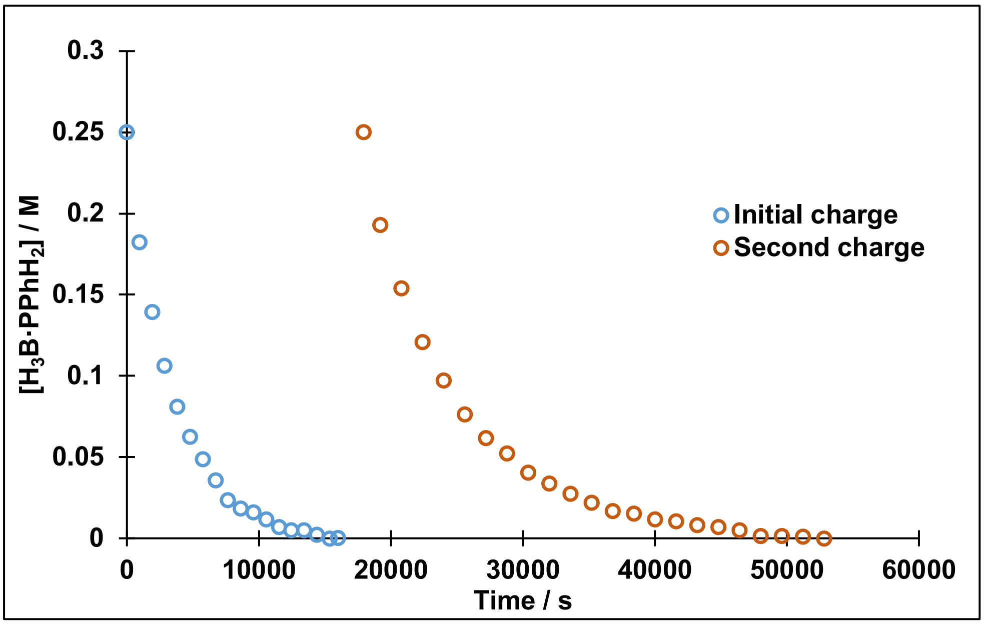

Figure S20. Concentration plot of $H_3B{\cdot}PPhH_2$ during the dehydropolymerisation of $H_3B{\cdot}PPhH_2$ using the precatalyst [Rh(dppe)$_2$]Cl with a re-charge of the same amount of monomer after 4.5 hours (3 mol%, 0.25 M in toluene-D$_8$, 373 K). Concentrations determined from the signal integrals from the in-situ monitored ^{1}H NMR spectra and are relative to the initial monomer concentration.

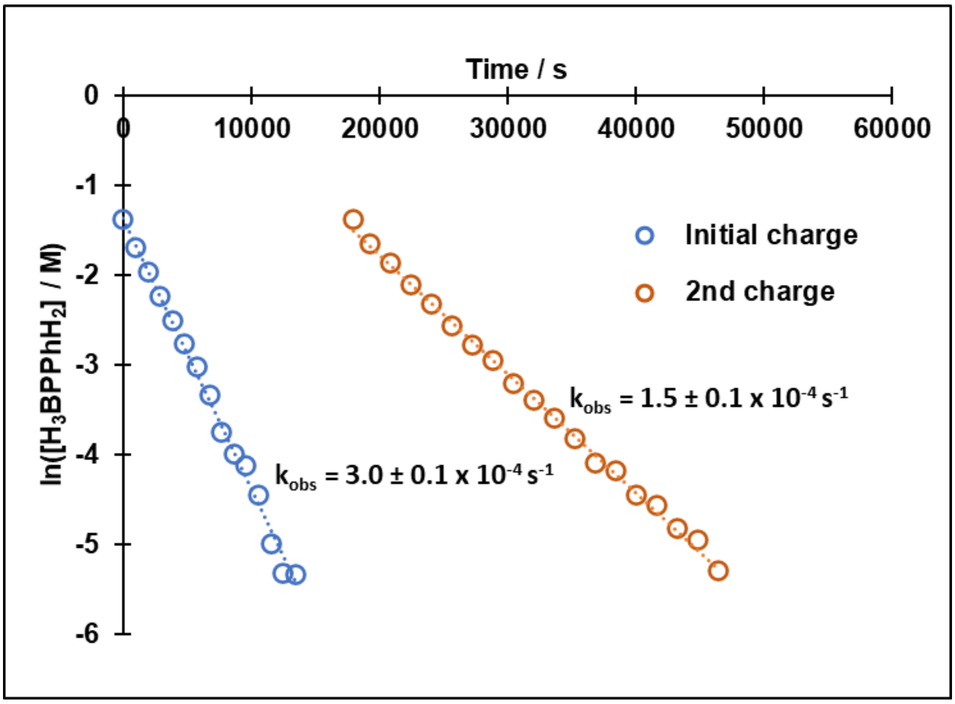

Figure S21. First order integrated rate plot of the re-charge experiment of the dehydropolymerisation of $H_3B{\cdot}PPhH_2$ (0.25 M, 3 mol% [Rh(dppe)$_2$]Cl, 100 °C). Concentrations determined from the signal integrals from the in-situ monitored ^{1}H NMR spectra and are relative to the initial monomer concentration.

Linear dimer ($H_3B{\cdot}PPhHBH_2{\cdot}PPhH_2$) preparation and reactivity studies

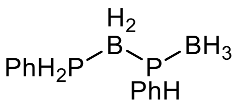

In a method similar to that previously reported with [RhCp*Me(PMe$_3$)CH$_2$Cl$_2$)][BAr$^F{}_4$][9] toluene (1.3 ml) was added to a flask containing [Rh(dppe)$_2$]Cl (15 mg, 0.016 mmol) and $H_3B{\cdot}PPhH_2$ (200 mg, 1.6 mmol). The flask was sonicated for five minutes before being heated to 100 °C with aid of an oil bath and left at this temperature, without stirring, for 45 minutes. The mixture was cooled to room temperature and the solvent was then removed *in vacuo*, resulting in a colourless residue. Hexane (5 ml) was added to the residue before sonication and then solvent was removed *in vacuo* to remove all the toluene. Hexane (10 ml) was added to the residue and the flask was sonicated for 10 minutes. The solvent mixture was removed by cannula transfer and the colourless solids were washed with hexane (10 ml) two further times and the hexane fractions were combined. The hexane solvent was removed *in vacuo* to leave a combination of $H_3B{\cdot}PPhH_2$ and $H_3B{\cdot}PPhHBH_2{\cdot}PPhH_2$ as an oily residue. $PhPH_2{\cdot}BH_3$ was removed by careful sublimation with a cold finger at -78 °C (45 °C, 5 x 10^{-2} mBar, 15 minutes) and the linear dimer was then diluted in toluene to form a stock solution. A small amount of $(H_3B)_2{\cdot}$dppe was also still present. The ^{31}P, ^{1}H and ^{11}B NMR spectroscopic signals were in good agreement with those previously reported.[9]

Linear dimer was then used as the monomer in catalytic conditions. Because only a small amount of the dimer could be obtained (17 mg) the concentration was reduced to 0.14 M. This reaction was also monitored by in-situ ^{1}H NMR spectroscopy (see below). This showed that polymer was indeed formed when linear dimer was used as “monomer” unit. The final polymer formed after 16 hours 100 °C was bimodal, comprising higher molecular polymer (M_n = 61,000 g mol^{-1}, PDI = 1.3) and lower molecular weight (M_n = 3,000 g mol^{-1}, PDI = 1.3) this is consistent with lower concentrations leading to more lower molecular weight polymer forming, as seen with $H_3B{\cdot}PPhH_2$. When $H_3B{\cdot}PPhH_2$ was catalysed at this lower concentration of 0.14 M in the same conditions the final polymer was also bimodal with calculated M_n values of 45,000 g mol^{-1} (1.3) and 2,500 g mol^{-1} (1.4).

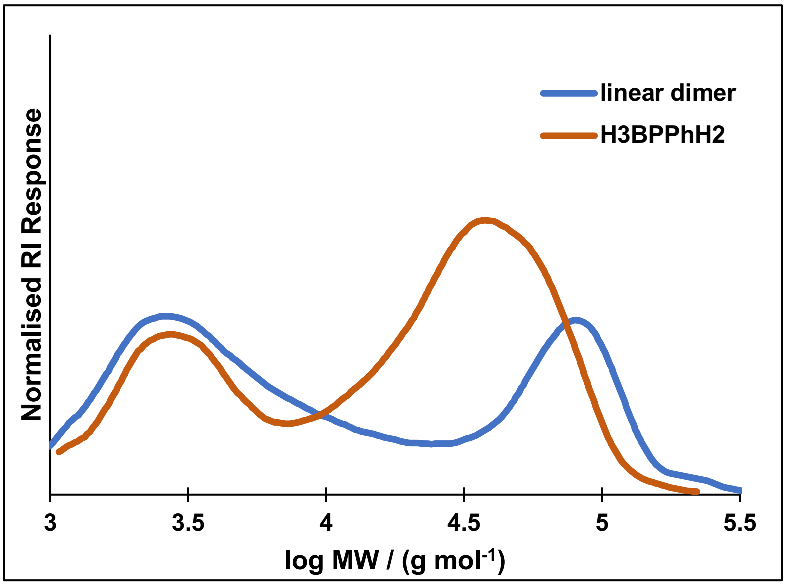

Figure S22. GPC trace of the poly(phenylphosphinoborane) formed from the dehydropolymerisation of $H_3B{\cdot}PPhH_2$ or $H_3B{\cdot}PPhHBH_2{\cdot}PPhH_2$ using [Rh(dppe)$_2$]Cl as a precatalyst (0.14 “monomer”, toluene, 100 °C, 3 mol% [Rh(dppe)$_2$]Cl).

The in-situ ^{1}H NMR spectrum closely resembles the reaction when $H_3B{\cdot}PPhH_2$ is used as the monomer. Free $PPhH_2$ and $H_{2(sol)}$ are observed and linear dimer is consumed as polymer formation occurs. In the hydride region, almost identical catalyst speciation is also observed. The regularly occurring signals at –1.8 and –14.5 in a 3:1 ratio, assigned as **2** are observed at the start of the reaction, which converts to a second species as the reaction progresses.

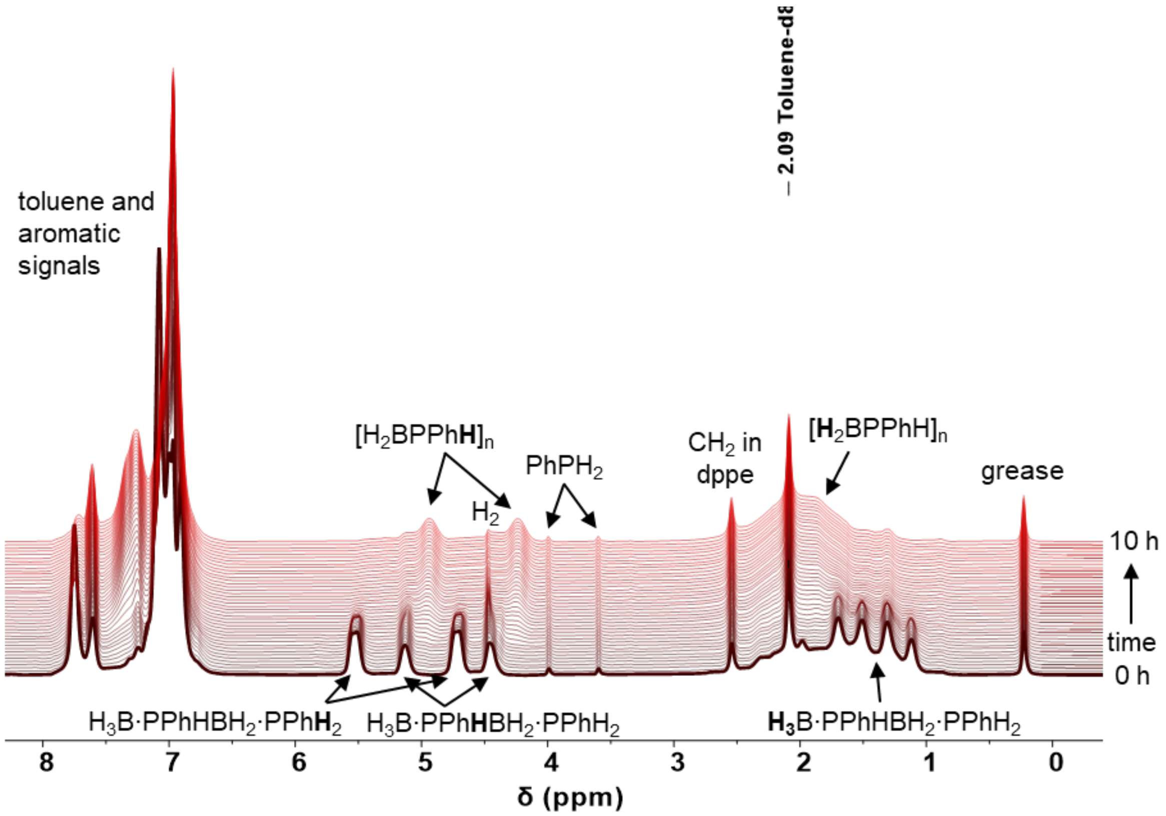

Figure S23. In-situ ^{1}H NMR of the dehydropolymerisation of $H_3B{\cdot}PPhHBH_2{\cdot}PPhH_2$, catalytic conditions: 3 mol% $[Rh(dppe)_2]Cl$, 0.14 M linear dimer in toluene-D_8, 373 K (500 MHz). Darker lines are earlier in the experiment and lighter red lines are at the end of the 10-hour experiment.

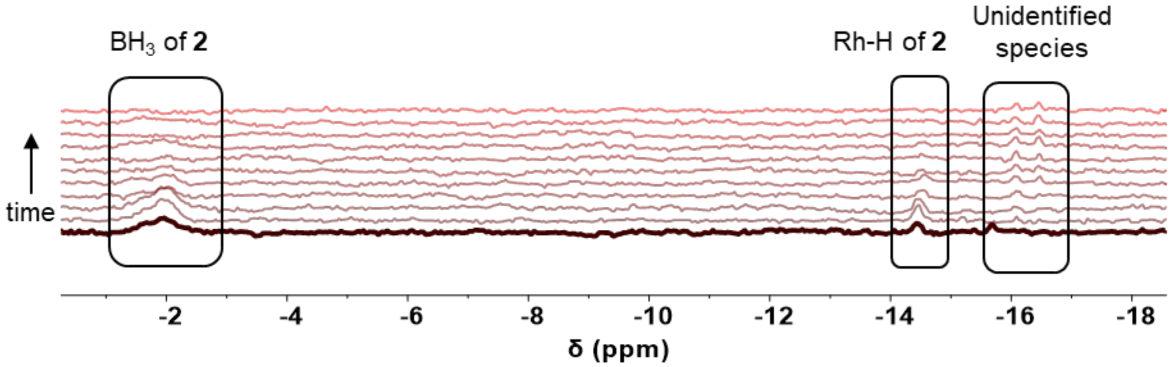

Figure S24. Hydride region of the in-situ ^{1}H NMR of the dehydropolymerisation of $H_3B{\cdot}PPhHBH_2{\cdot}PPhH_2$, catalytic conditions: 3 mol% $[Rh(dppe)_2Cl]$, 0.14 M linear dimer in toluene-D_8, 373 K (500 MHz). Darker lines are earlier in the experiment and lighter red lines are at the end of the 10-hour experiment.

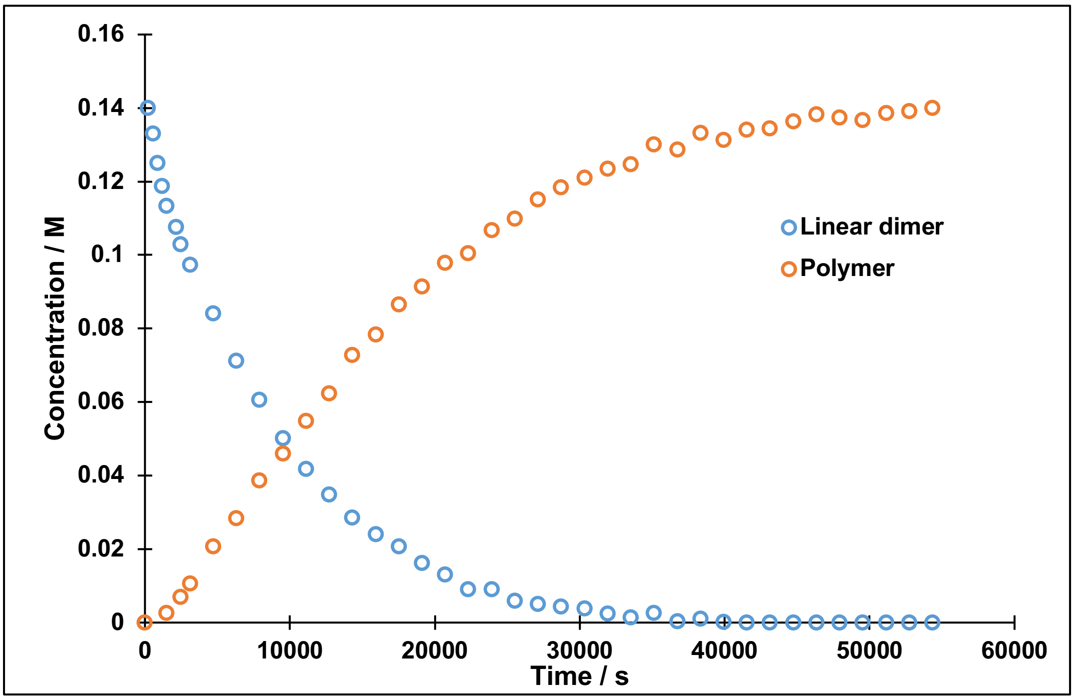

Figure S25. Concentration plot of linear dimer ($H_3B{\cdot}PPhHBH_2{\cdot}PPhH_2$) and polymeric $[H_2BPPhH]_n$ during the dehydropolymerisation of $H_3B{\cdot}PPhHBH_2{\cdot}PPhH_2$ using $[Rh(dppe)_2]Cl$ as a precatalyst (3 mol%, 0.14 M in toluene-D_8, 373 K). Concentrations determined from the signal integrals in the in-situ monitored ^1H NMR spectra and are relative to the initial monomer concentration.

The dehydropolymerisation of $H_3B{\cdot}PPhH_2$ was repeated at 0.14 M to compare the rate of reaction between $H_3B{\cdot}PPhH_2$ and $H_3B{\cdot}PPhHBH_2{\cdot}PPhH_2$. In the plot below, it is evident that $H_3B{\cdot}PPhH_2$ is consumed faster at the start of the reaction but they ultimately both require a similar time for complete conversion.

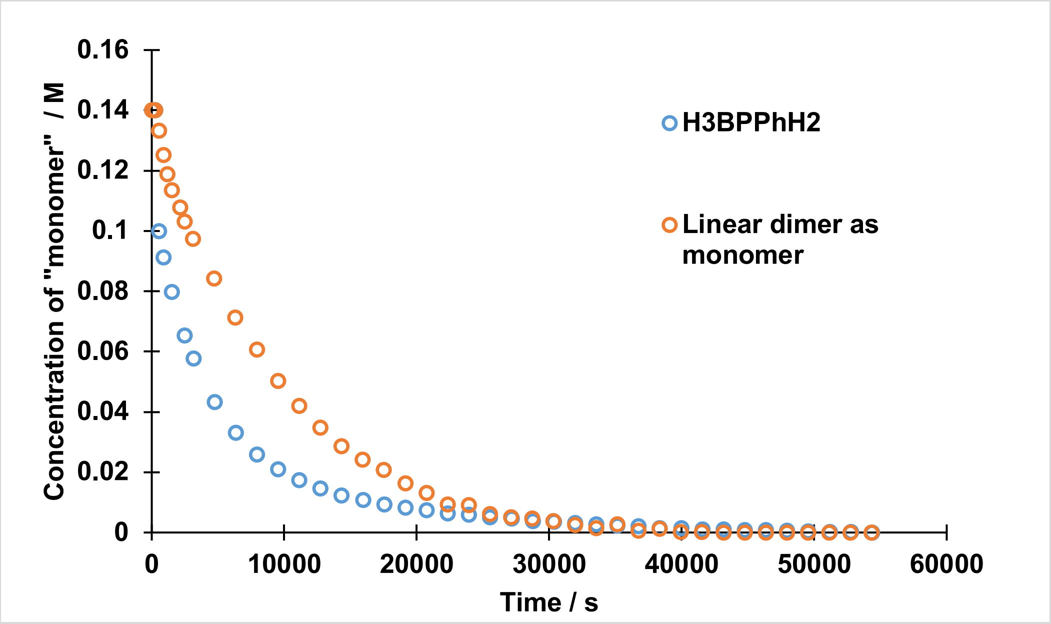

Figure S26. Concentration plot of $H_3B{\cdot}PPhH_2$ and $H_3B{\cdot}PPhHBH_2{\cdot}PPhH_2$ during the dehydropolymerisation using $[Rh(dppe)_2]Cl$ as a precatalyst (3 mol%, 0.14 M in toluene-D_8, 373 K). Concentrations determined from the signal integrals from the in-situ monitored ^1H NMR spectra and are relative to the initial monomer concentration.

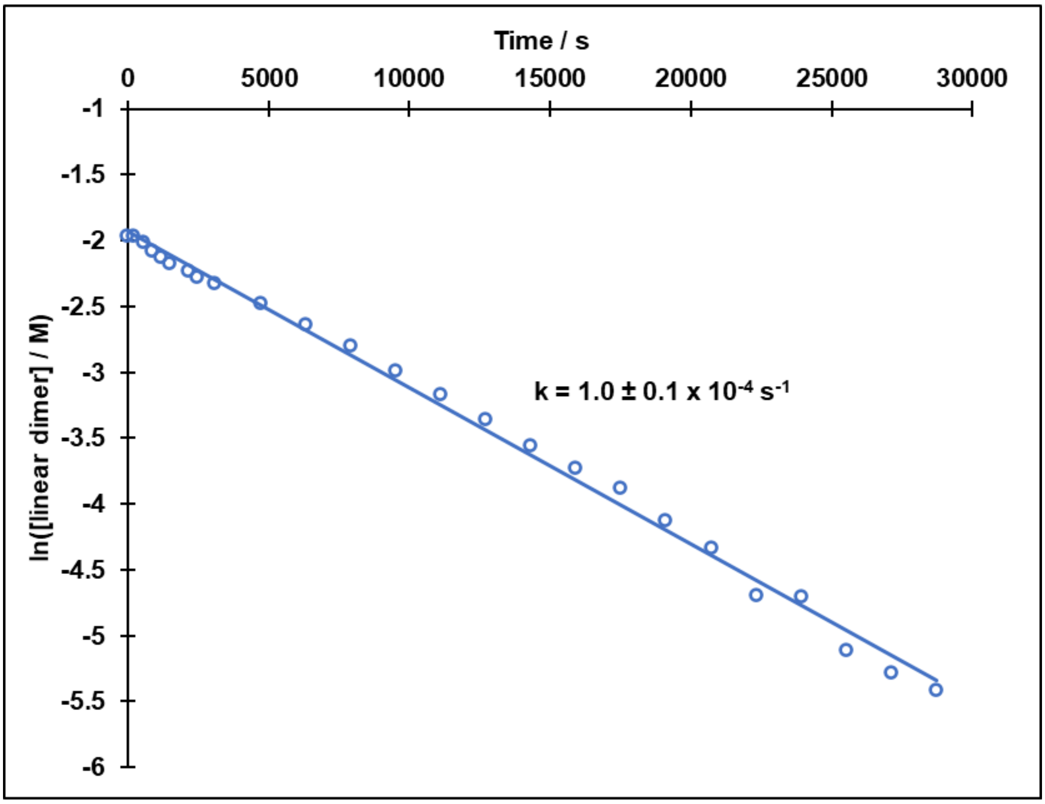

Figure S27. First order integrated rate plot the dehydropolymerisation of $H_3B{\cdot}PPhHBH_2{\cdot}PPhH_2$ (0.14 M, 3 mol% [Rh(dppe)$_2$]Cl, 100 °C). Concentrations determined from the signal integrals from the in-situ monitored ^{1}H NMR spectra and are relative to the initial $H_3B{\cdot}PPhHBH_2{\cdot}PPhH_2$ concentration.

Speciation and stoichiometric experiments

Through a number of stoichiometric experiments, detailed below, the linear dimer bound complex, [Rh(dppe)H(σ,η-PPhHBH$_2$PPhHBH$_3$)]Cl (**2**) is assigned in-situ with the following data which matches with the in-situ catalyst speciation observations.

^{1}H NMR (500 MHz, toluene-D$_8$, 298 K) selected data: δ 2.09 (s, 4H, PPh$_2$C*H*$_2$C*H*$_2$PPh$_2$), –1.9 (br m, 3H, Rh–H$_3$B), –14.2 (br s, 1H, Rh–H).

^{11}B{^{1}H} NMR (160 MHz, toluene-D$_8$, 298 K) selected data: δ –16.7 (br s, BH$_2$) and –3.2 (br s, BH$_3$).

ESI-MS (1,2-F$_2$C$_6$H$_4$): *m/z* [M]$^+$ 747.2 (Calc. 747.2) with the correct isotope pattern.

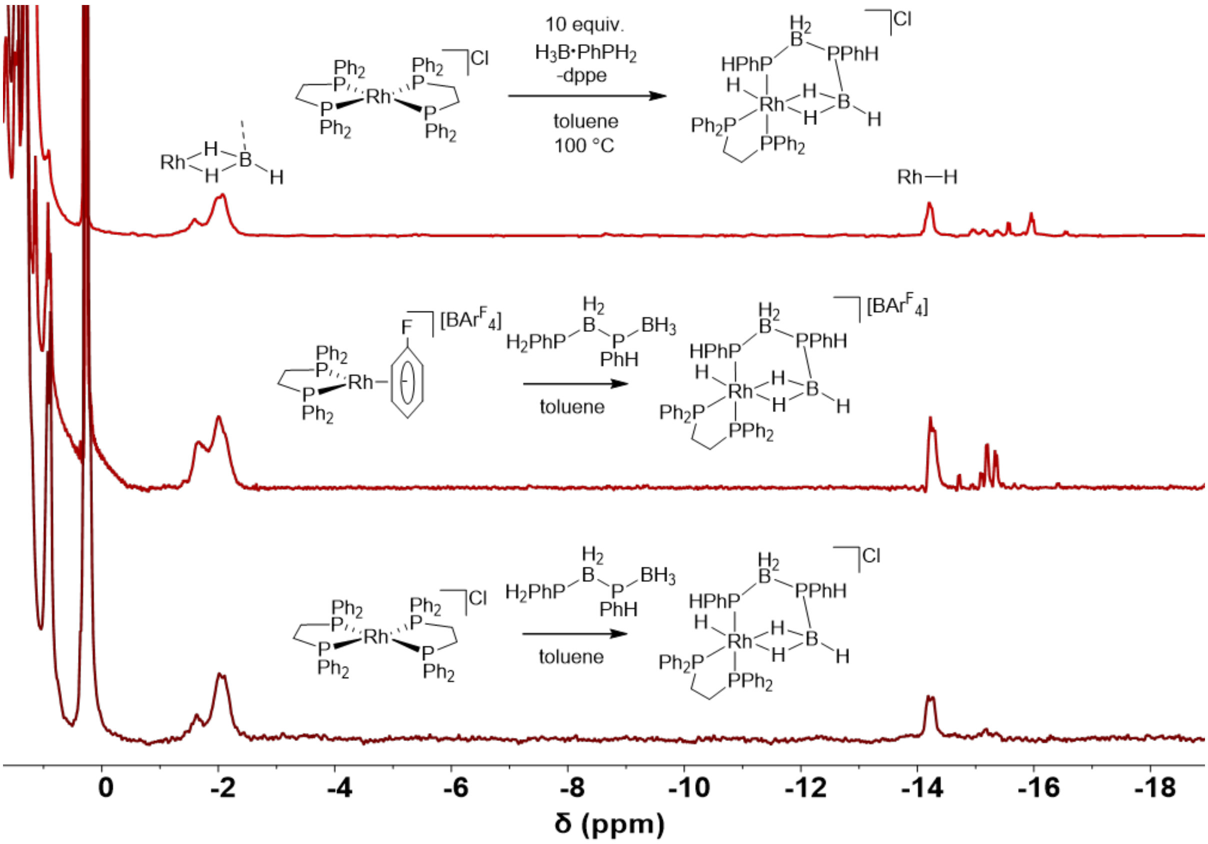

Figure S28. The hydride region of the ^{1}H NMR spectra of speciation experiments which support the formation of **2** during catalysis (600 MHz, toluene-D_8, 298 K).

To a high pressure NMR tube was added $[Rh(dppe)_2]Cl$ (7.5 mg, 0.008 mmol), $H_3B{\cdot}PPhH_2$ (10 mg, 0.08 mmol) and toluene-D_8 (0.32 ml, 0.25 M). The NMR tube was sonicated for five minutes before being inserted into an oil bath at 100 °C for 10 minutes (start of catalysis) after which the NMR tube was washed with CH_2Cl_2 and the mixture observed via NMR spectroscopy.

After 10 minutes, 30% of $H_3B{\cdot}PPhH_2$ was converted. The ^{1}H NMR spectra reveals hydride signals very similar to those observed in the sin-situ NMR studies at 1 mol% catalyst loading (δ –2.0, 3H, BH_3 and δ –14.2, 1H, Rh-H) which are attributed to a Rh(**III**) P-H activated, linear dimer bound complex **2**. The BH_3 signal sharpens upon ^{11}B-decoupling and is split into two, which could be an effect of the diastereomeric nature of the P atoms,[10] but this is not observed in the Rh-H signal. There is also some evidence of an unknown complex appearing with hydride signals at δ –15 to –16. Signals from oligomeric/polymeric materials pollutes the clarity of the rest of the ^{1}H NMR spectrum. The $^{31}P\{^{1}H\}$ NMR spectrum is broad and not useful for structural elucidation. Broad signals at δ –16.7 and –3.2 in the $^{11}B\{^{1}H\}$ NMR spectrum are consistent with the BH_2 and coordinated BH_3 respectively, concurrent with the similarly reported complex; $[Rh(dppp)H(\sigma,\eta^2\eta^2\text{-}PPh_2BH_2PPh_2BH_3)][BAr^F_4]$ (δ 3.2 and –27.2).[11]

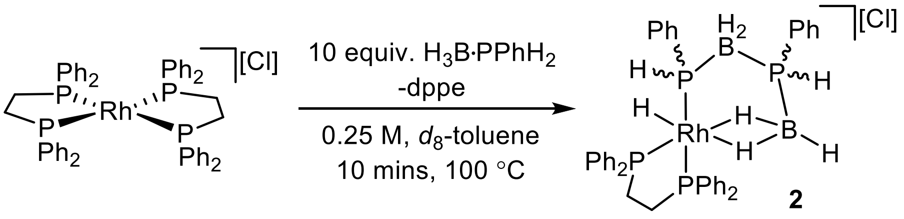

Scheme S2. Speciation experiment to ascertain the catalytic resting state of $[Rh(dppe)]^{+}$ catalysed $H_3B{\cdot}PPhH_2$ dehydropolymerisation.

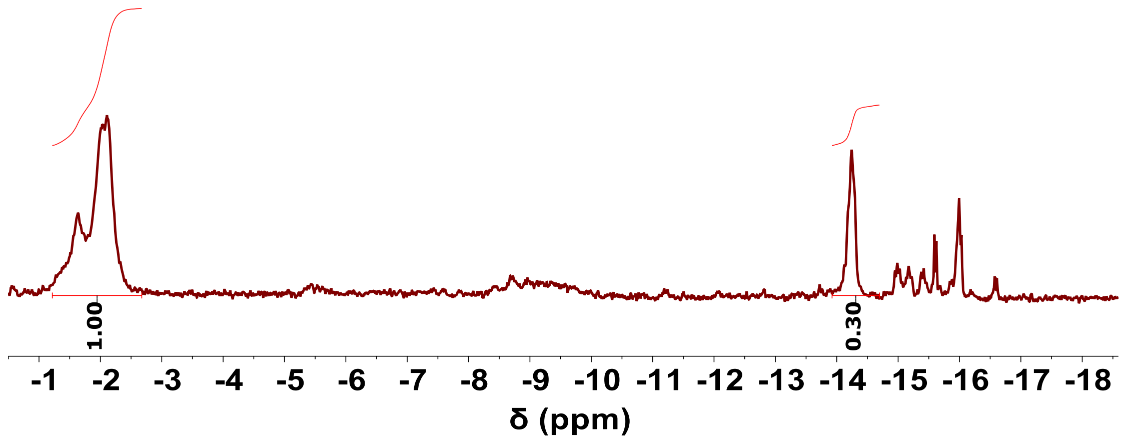

Figure S29. ^{1}H NMR spectrum of the catalytic mixture of $H_3B{\cdot}PPhH_2$ with 10 mol% $[Rh(dppe)_2]Cl$ in toluene (0.25 M) stopped after 10 minutes at 100 °C (500 MHz, Toluene-D_8, 298 K).

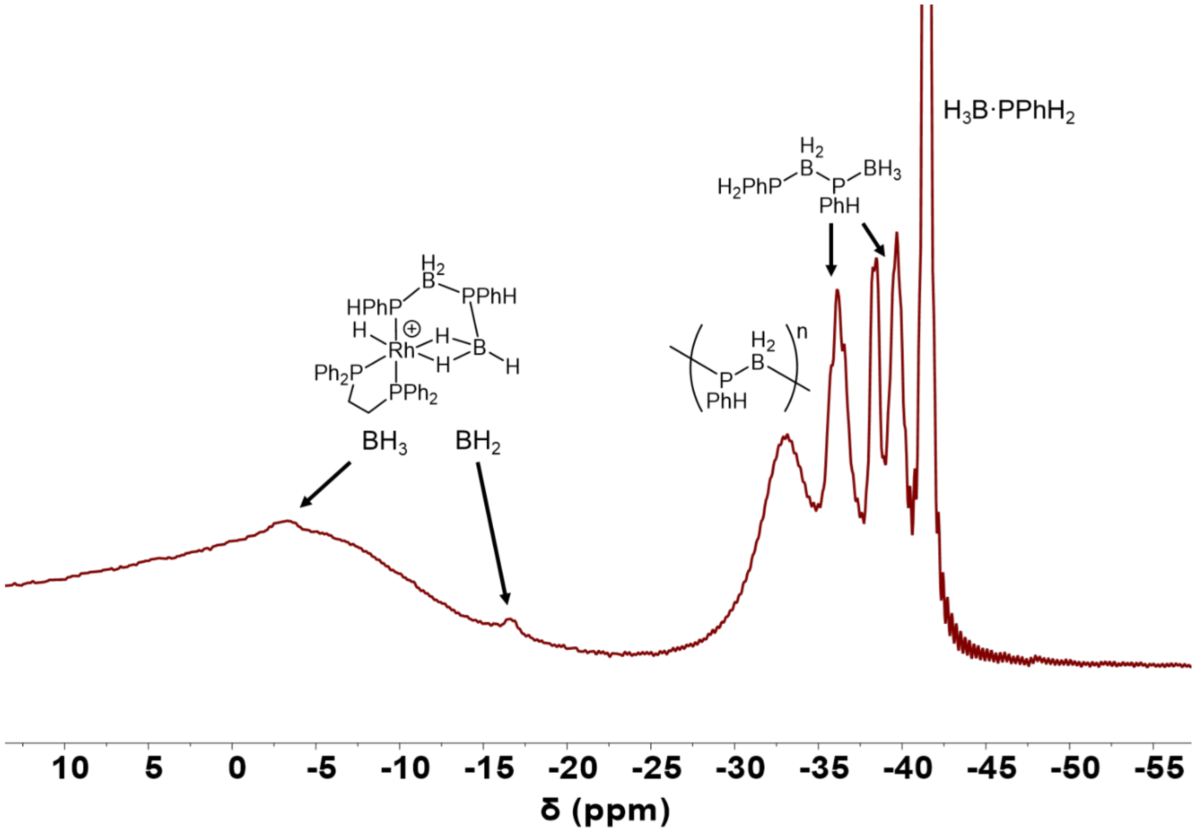

Figure S30. ^{11}B{^{1}H} NMR spectrum of the catalytic mixture of $H_3B{\cdot}PPhH_2$ with 10 mol% $[Rh(dppe)_2]Cl$ in toluene (0.25 M) stopped after 10 minutes at 100 °C (160 MHz, toluene-D_8, 298 K).

To further support the theory of a Rh(III) P–H activated catalyst resting state at the start of catalysis, **2**, stoichiometric reactivity studies were conducted. Firstly, free linear dimer was added to the precatalyst $[Rh(dppe)_2]Cl$. To a Youngs NMR tube was added $[Rh(dppe)_2]Cl$ (5 mg, 0.005 mmol), $H_3B{\cdot}PPhHBH_2{\cdot}PPhH_2$ (4 mg, 0.015 mmol) and toluene-D_8 (0.5 ml) and the NMR tube was heated for five minutes at 100 °C. The NMR tube was allowed to cool to room temperature and cleaned with CH_2Cl_2 and the reaction mixture observed by NMR spectroscopy. In the hydride region of the ^{1}H NMR spectrum, the same hydride signals at δ – 2.0 and –14.2 in a 3:1 ratio were observed. Moreover, when dppe (4 mg, 0.01 mmol) was added into the reaction mixture all of the hydride signals disappeared, and $[Rh(dppe)_2]Cl$ was reformed, illustrating the reversibility of this reaction.

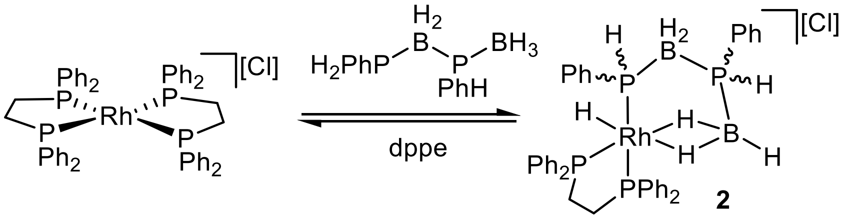

Scheme S3. Addition of linear dimer ($H_3B{\cdot}PPhHBH_2{\cdot}PPhH_2$) to $[Rh(dppe)_2]Cl$ in toluene-D_8. Addition of dppe to the linear dimer bound complex reformed $[Rh(dppe)_2]Cl$.

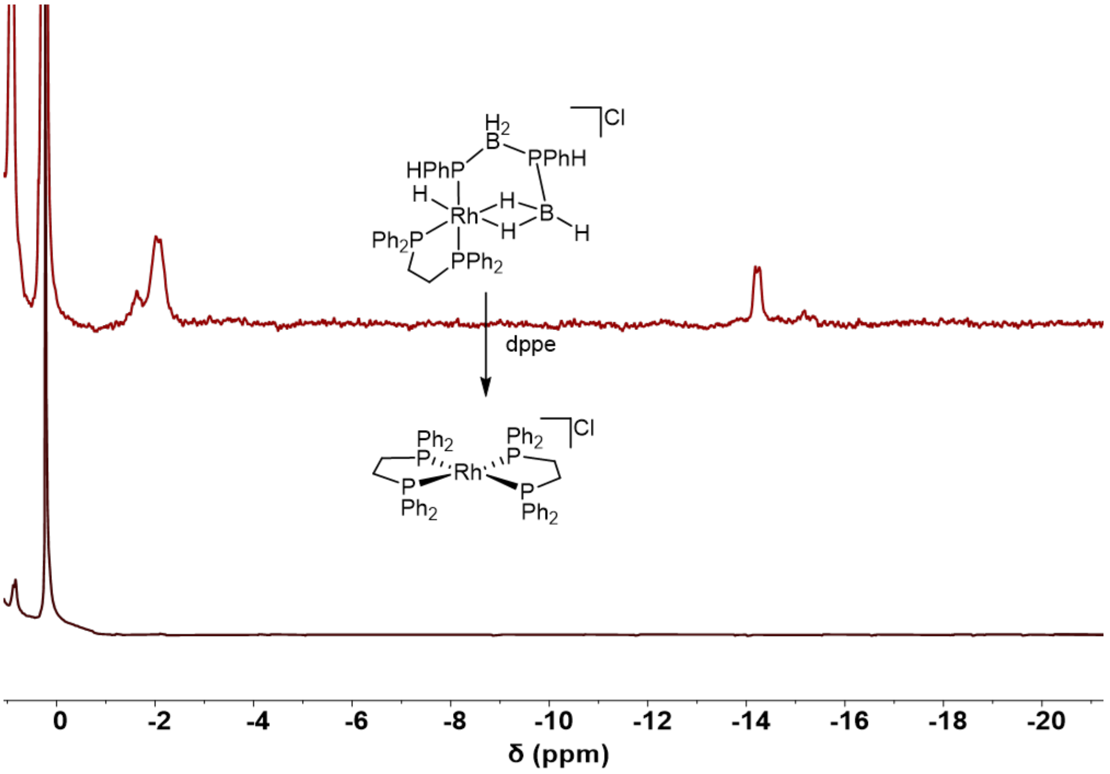

Figure S31. Hydride region of **2** formed from the addition of free linear dimer to $[Rh(dppe)_2]Cl$ in toluene-D_8 and then addition of dppe to reform $[Rh(dppe)_2]Cl$.

Free linear dimer was also added to a more active Rh complex: $[Rh(dppe)(\eta^6\text{-}C_6H_5F)][BAr^F{}_4]$, which was prepared via a literature procedure.[12] To a Youngs NMR tube was added $[Rh(dppe)(\eta^6\text{-}C_6H_5F)][BAr^F{}_4]$ (15 mg, 0.01 mmol), $H_3B{\cdot}PPhHBH_2{\cdot}PPhH_2$ (5 mg, 0.02 mmol) and toluene-D_8 (0.5 ml) and the NMR tube was sonicated for five minutes before the mixture was investigated by NMR spectroscopy. Once again, hydride signals corresponding to **2** were observed as two sets of hydride signals corresponding to the coordinated BH_3 (δ –1.8) and Rh-H (δ –14.2) in the ^{1}H NMR spectrum. Multiple signals are observed in both regions. In the ^{31}P{^{1}H} NMR spectrum, broad signals between δ 65-50 are likely signals corresponding to the dppe portion of **2** and the broad signals between δ –35 and –45 could be the P-B ligand. Low temperature NMR was explored but no further structural information was obtained. The ESI mass spectrum shows that the major species has a mass m/z of 747.2, this is in good agreement with the theoretical value for the cationic portion of **2** of 747.2 m/z and the isotope distributions match well. Some $[Rh(dppe)_2]^+$ is also observed in the mass spectrum which is carried over from the Rh precursor.

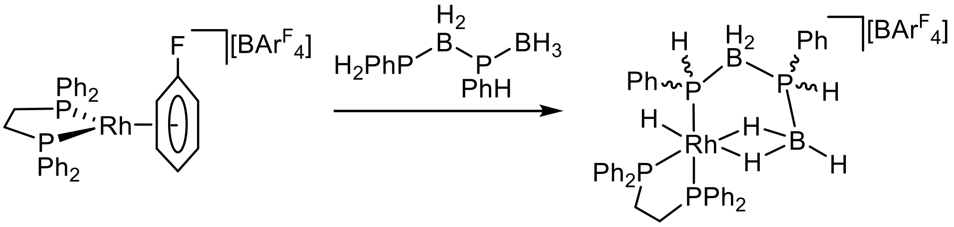

Scheme S4. Addition of $H_3B{\cdot}PPhHBH_2{\cdot}PPhH_2$ to [Rh(dppe)(η^6-C_6H_5F)][BAr$^F{}_4$] in toluene-D_8 to form **2**.

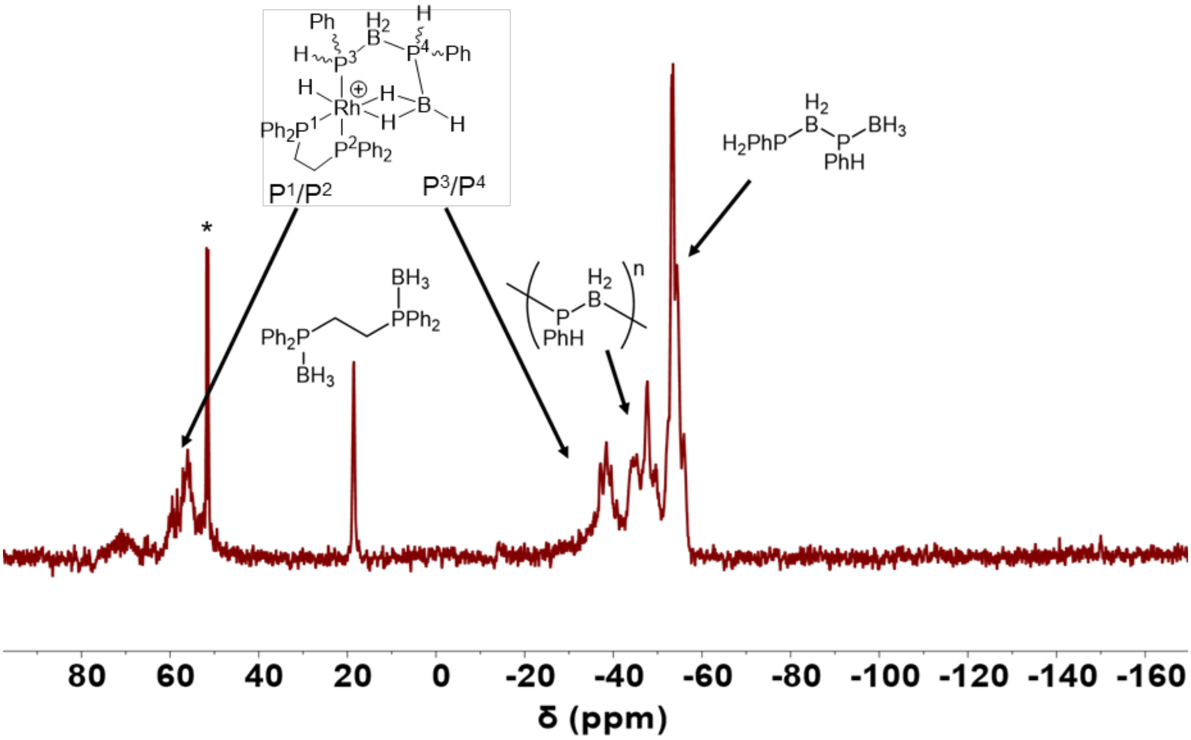

Figure S32. $^{31}P\{^1H\}$ NMR spectrum of the reaction mixture of [Rh(dppe)(η^6-C_6H_5F)][BAr$^F{}_4$] and $H_3B{\cdot}PPhHBH_2{\cdot}PPhH_2$ in Toluene-D_8 (243 MHz, Toluene-D_8, 298 K). * Denotes unknown impurity.

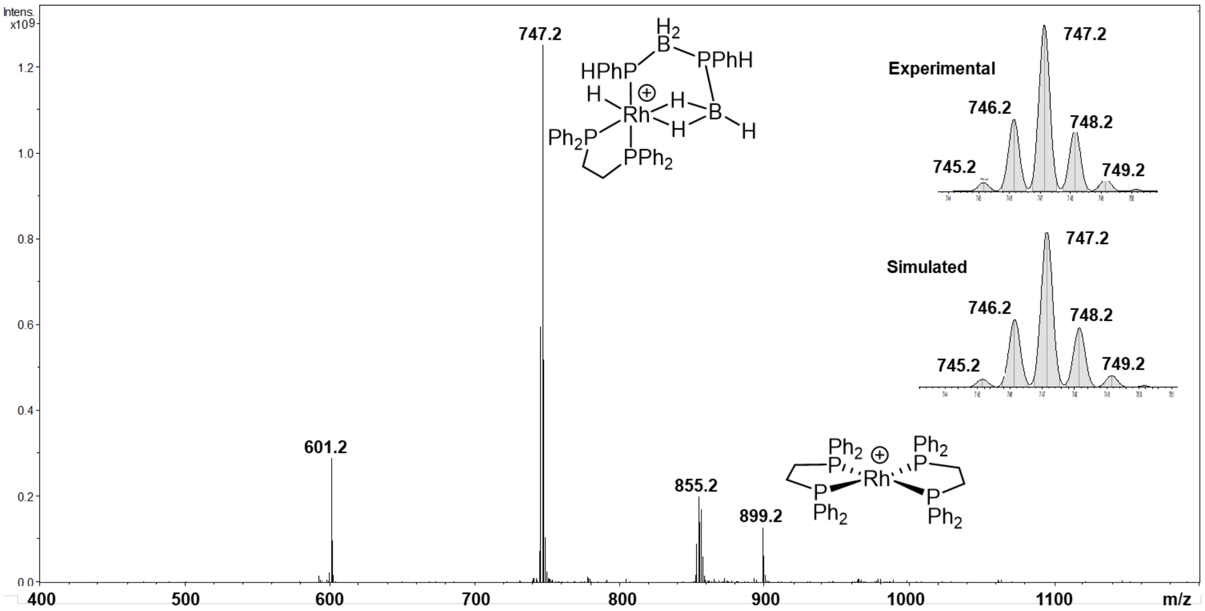

Figure S33. ESI-MS (positive mode) mass spectrum of the reaction mixture of [Rh(dppe)(η^6-C_6H_5F)][$BAr^F{}_4$] and $H_3B{\cdot}PPhHBH_2{\cdot}PPhH_2$. Inset shows the experimental and simulated isotope distribution of the cationic portion of **2**.

Mechanism support – dehydrocoupling of oligomeric [H_2BPPhH]$_n$

Oligomeric [H_2BPPhH]$_n$ was formed via the general polymer formation procedure (page S3) with a reaction time of four hours {1 mol% [Rh(dppe)$_2$]Cl, 1.25 M $H_3B{\cdot}PPhH_2$, toluene}. The polymer was isolated, and the M_n was measured by GPC analysis to be 6,000 g mol^{-1} with a wide polydispersity (Đ = 2.8). The isolated polymer (31 mg) was redissolved in toluene (0.2 ml) and added to fresh catalyst [Rh(dppe)$_2$]Cl (0.23 mg) and the was mixture heated to 100 °C and left for 19 hours. The reaction mixture was cannula transferred into a Youngs flask and hexane was added, resulting in the formation of a white precipitate. The solvent was removed and the solid washed with hexane before it was dried under Schlenk line vacuum (< 1 x 10^{-1} mBar). The molecular weight of the new polymer was determined to be 49,000 g mol^{-1} with a narrower polydispersity of 1.5. This is further evidence for the proposed reversible chain transfer mechanism. In the GPC trace, shorter molecular weight species, possibly cyclic species, have not reacted further and could not be washed away with hexane. Cyclic polymers are more commonly found in step-growth like polymerisations.[13]

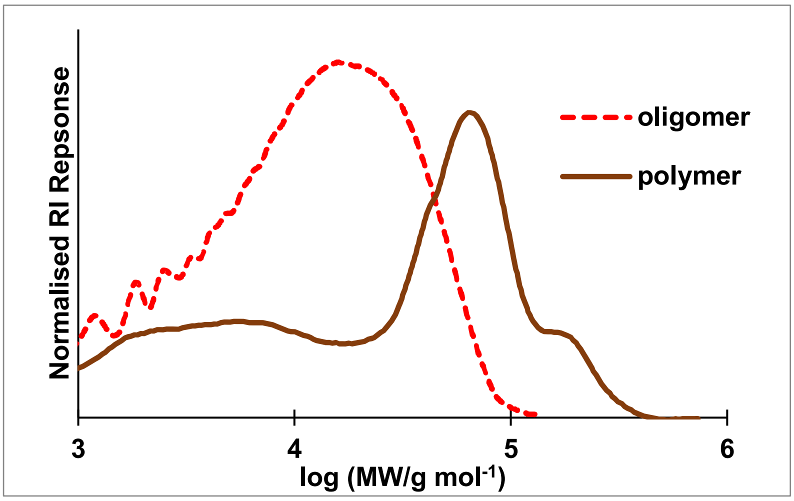

Figure S34. GPC traces of oligomeric $[H_2BPPhH]_n$ produced from the dehydropolymerisation of $H_3B{\cdot}PPhH_2$ using $[Rh(dppe)_2]Cl$ as a precatalyst (1 mol%, toluene, 1.25 M, 100 °C) after four hours of reaction time (red) and polymeric $[H_2BPPhH]_n$ produced via the dehydropolymerisation of oligomeric $[H_2BPPhH]_n$ with a 19 hour reaction time (brown).

MALS GPC

The polymer molecular weights discussed so far have been determined by conventional column calibration (CCC) using an RI detector and polystyrene calibration samples on a GPC instrument. For comparison, a MALS detector was also used on the same instrument to determine the absolute molecular weight. This showed that the CCC method overestimated the polymer molecular weight by roughly 3-4 times at higher molecular weight and up to 8 times at lower molecular weight. However, MALS can only measure polymer of M_n > 4000 g mol-1 (by MALS) and therefore a combination of the two methods is required to analysed the shorter and longer chain polymers form in this report. Below is a calibration graph between the two analysis methods.

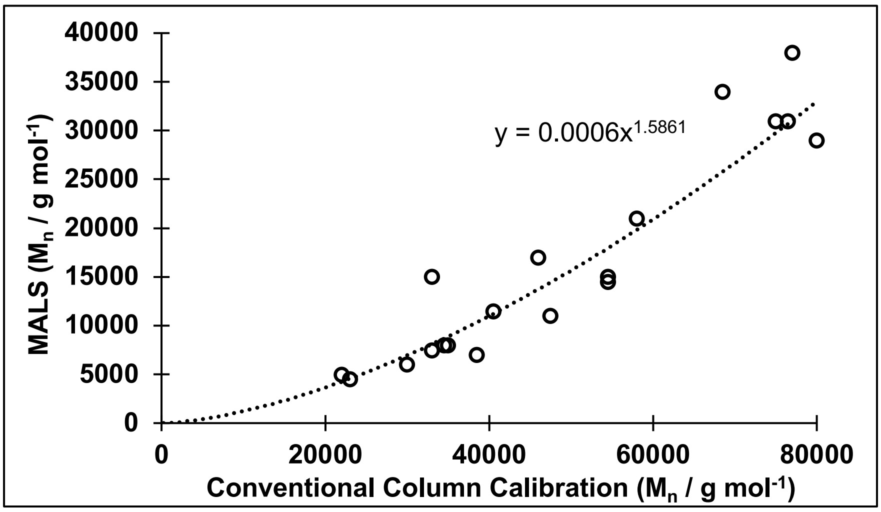

Figure S35. Calibration graph of the GPC analysis of samples of poly(phenylphosphinoborane), obtained via the dehydropolymerisation of $H_3B{\cdot}PPhH_2$ using $[Rh(dppe)_2]Cl$ as a precatalyst, between conventional column calibration (using a polystyrene calibration) and a multi-angle light scattering detector (MALS).

Formation of $[H_2BPPhH]_{110}$-*b*-$[H_2BP(n\text{-}hex)H]_{11}$ BCP1

A copolymer of poly(phenylphosphinoborane) and poly(n-hexylphosphinoborane) was produced via the addition of $H_3BP(n\text{-}hex)H_2$ to preformed and isolated $[H_2BPPhH]_n$, which was formed via the general procedure (page S3) and a reaction time of 19 hours. $H_3BP(n\text{-}hex)H_2$ (51 mg, 0.375 mmol, 1.25 M), $[Rh(dppe)_2]Cl$ (10.5 mg, 0.021 mmol, 3 mol%), $[H_2BPPhH]_n$ (30 mg, M_n = 28,500 g mol^{-1}, Đ = 1.3) and toluene (0.2 ml) were added to a high pressure NMR tube. The NMR tube was sonicated for five minutes before being heated to 100 °C in an oil bath and maintained at this temperature for 66 hours. The colourless reaction mixture was analysed by NMR spectroscopy, which revealed that all the $H_3BP(n\text{-}hex)H_2$ had reacted. Signals corresponding to $[H_2BP(n\text{-}hex)H]_n$, and $[H_2BPPhH]_n$ are observed in the $^{31}P\{^1H\}$ NMR spectrum. The reaction mixture was transferred via cannula to a vial, to which was added hexane (10 ml) and the mixture stirred vigorously for two hours. The solvent was removed, and the colourless residue was washed with further hexane (2 x 10 ml). The resultant colourless residue was dried under Schlenk line vacuum (< 1 x 10^{-1} mbar) overnight to yield 34 mg of **BCP1**. The polymer was characterised via multinuclear NMR spectroscopy, GPC and DLS.

^{31}P NMR (243 MHz, $CDCl_3$, 298 K): –49.4 (br d, J_{PH} = 358 Hz, $[H_2B\textit{P}PhH]_n$), –63.4 (br m, $[H_2B\textit{P}(n\text{-}hex)H]_n$).

$^{31}P\{^1H\}$ NMR (243 MHz, CD_2Cl_2, 298 K): δ –49.4 (br s, $[H_2B\textit{P}PhH]_n$), –63.4 (br m, $[H_2B\textit{P}(n\text{-}hex)H]_n$).

^{11}B NMR (193 MHz, CD_2Cl_2, 298 K): δ –35.5 (br m).

^{1}H NMR (600 MHz, CD_2Cl_2, 298 K): δ 7.68-6.69 (br m, 5H, $[H_2BP\textit{Ph}H]_n$), 4.27 (br d, J_{PH} = 358 Hz, 1H, $[H_2BPPh\textit{H}]_n$), 3.66 (br d, J_{PH} = ~340 Hz, 0.1H, $[H_2BP(n\text{-}hex)\textit{H}]_n$), 1.87-0.69 (br m, ~4H). This latter signal comprises the BH_2 signals of both polymer blocks (2.2H), the n-hexyl CH_2 signals (δ 1.56 and 1.28, 1H) and the CH_3 signal (δ 0.87, 0.3H).

The in-situ $^{31}P\{^1H\}$ spectrum after 66 hours at 100 °C reveals that all of the $H_3B{\cdot}P(n\text{-}hex)H_2$ has reacted and there is a mixture of and polymeric materials, lots of smaller side products, $(BH_3)_2$-dppe and $P(n\text{-}hex)H_2$ in the reaction mixture. However, washing the mixture with hexane cleaned up the mixture so that only three major signals are observed. The two broad signals at δ –49.4 and –63.4 are consistent with the ^{31}P signals for the homopolymers $[H_2BPPhH]_n$[6] and $[H_2BP(n\text{-}hex)H]_n$[7] respectively. The relative integrals of these signals is roughly 10:1. This matches well with the integral of the P–H signals in the ^{1}H NMR spectrum, which also integrate 10:1, and are also consistent with the reported shits for the homopolymers.[6, 7] A third signal is observed in the ^{31}P NMR spectrum at δ –56.2. This is in the region of oligomeric $[H_2BP(n\text{-}hex)H]_n$ species. The ^{11}B NMR signal is broad and observed at a similar chemical shift for the reported homopolymers (δ –30 to –42).[6, 7]

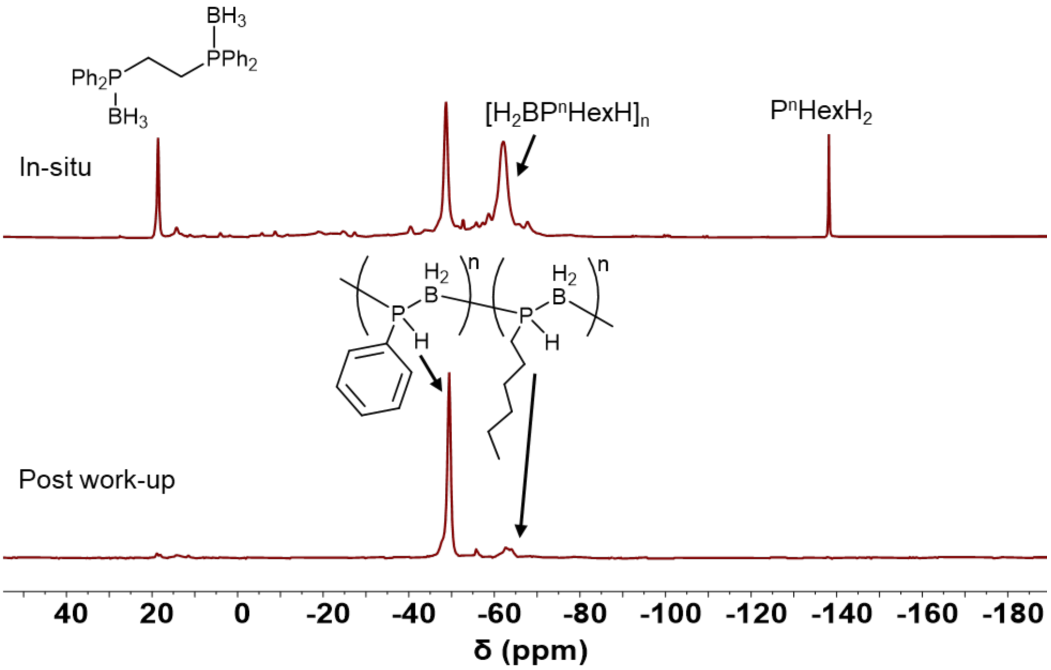

Figure S36. In-situ (top) and isolated (bottom) $^{31}P\{^{1}H\}$ NMR spectra of **BCP1** (243 MHz, toluene or $CDCl_3$ respectively).

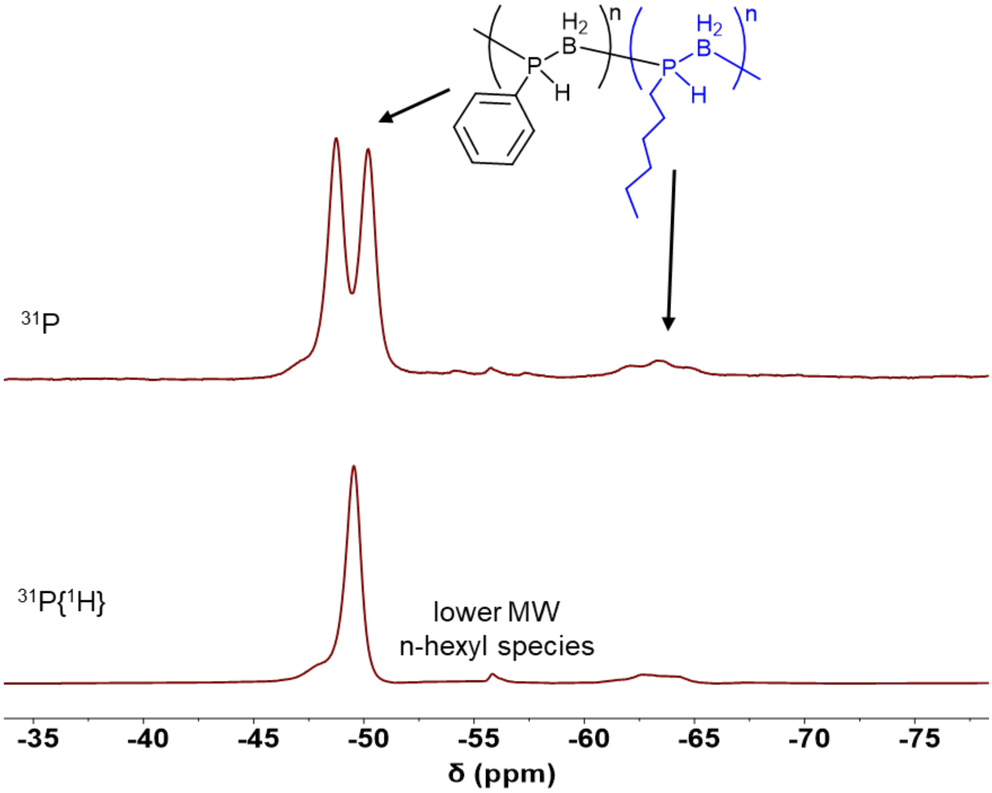

Figure S37. ^{31}P and $^{31}P\{^{1}H\}$ NMR spectra of **BCP1** (243 MHz, $CDCl_3$, 298 K).

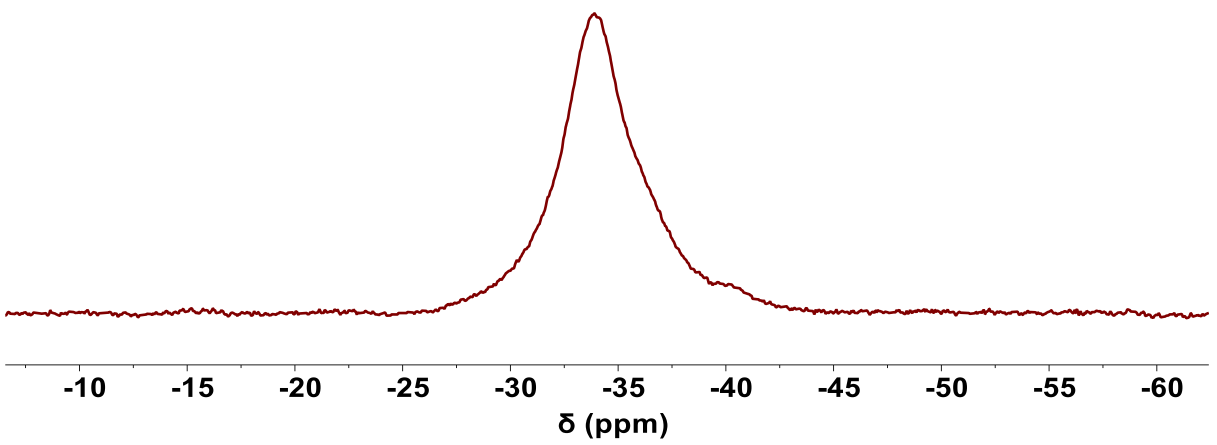

Figure S38. ^{11}B NMR spectrum of **BCP1** (193 MHz, $CDCl_3$, 298 K).

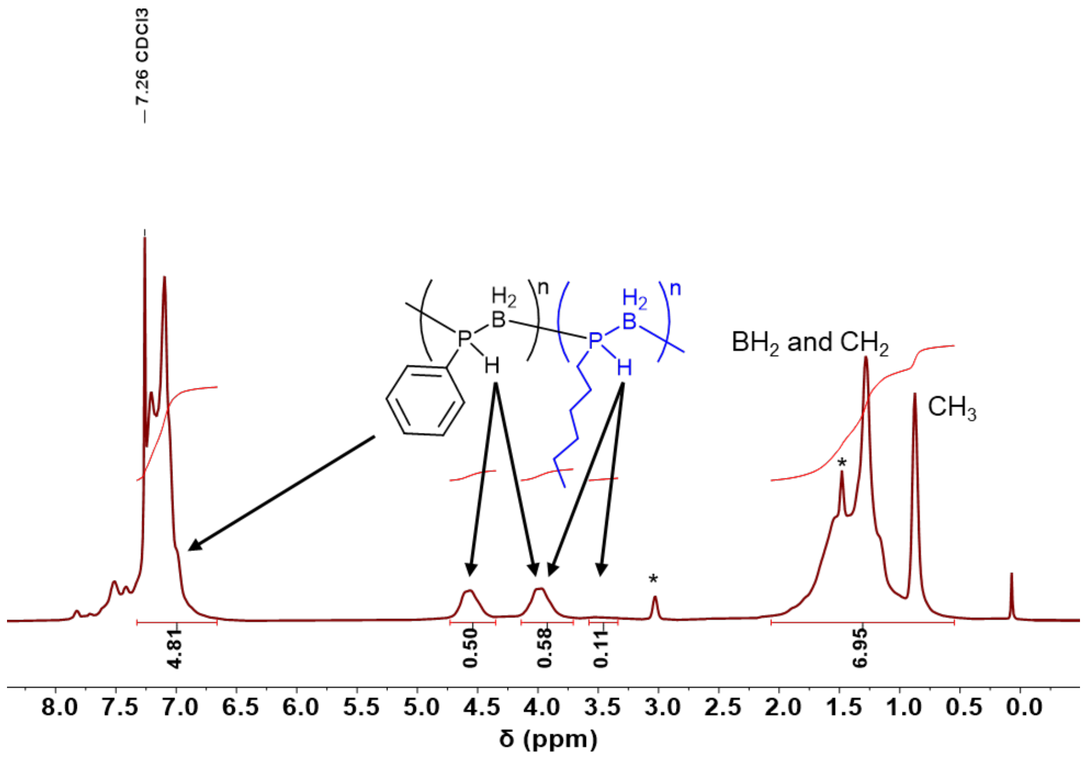

Figure S39. ^{1}H NMR spectra of **BCP1** (600 MHz, $CDCl_3$, 298 K). Integrals relative to the P-H signal in the $[H_2BPPhH]_n$ block. * Denotes unknown impurity.

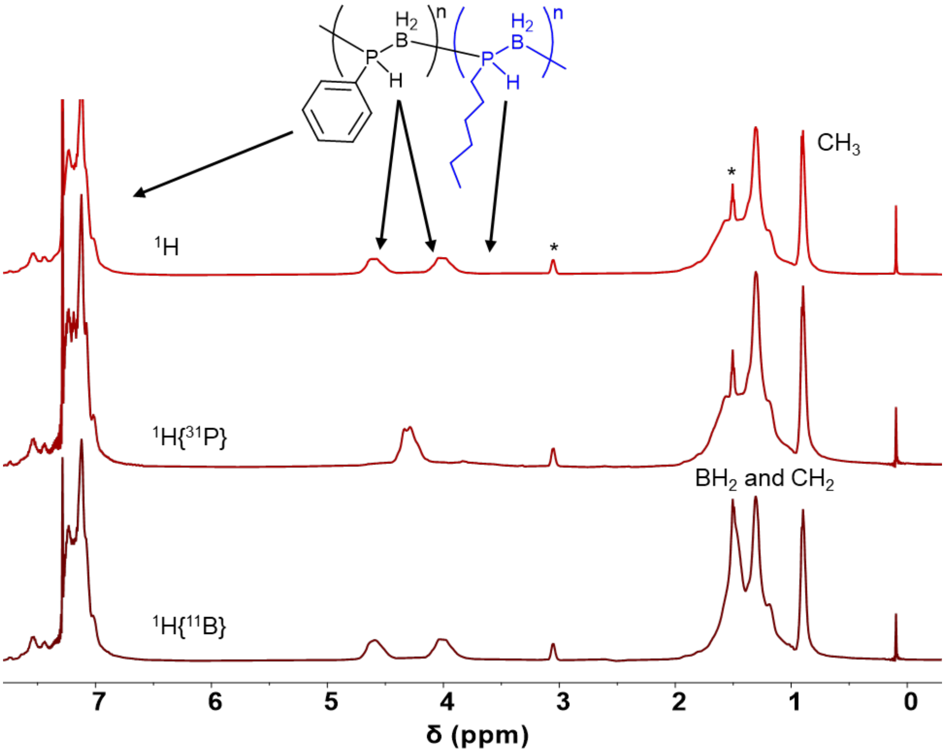

Figure S340. ^{1}H (top), $^{1}H\{^{31}P\}$ NMR (middle) and $^{1}H\{^{11}B\}$ (bottom) spectra of **BCP1** (600 MHz, $CDCl_3$, 298 K). * Denotes unknown impurity.

GPC Analysis

Table S4. GPC data for **BCP1**.

	**BCP1**
Ph:(n-hex)	10:1
M_n (g mol^{-1}) (CCC)	46,000[a]
Polydispersity (Đ) (CCC)	1.4[a]
M_n (g mol^{-1}) (MALS)	15,000
Polydispersity (Đ) (MALS)	1.3
Starting $[H_2BPPhH]_n$	28,500 (1.3)

[a] Measurement of the higher molecular weight portion.

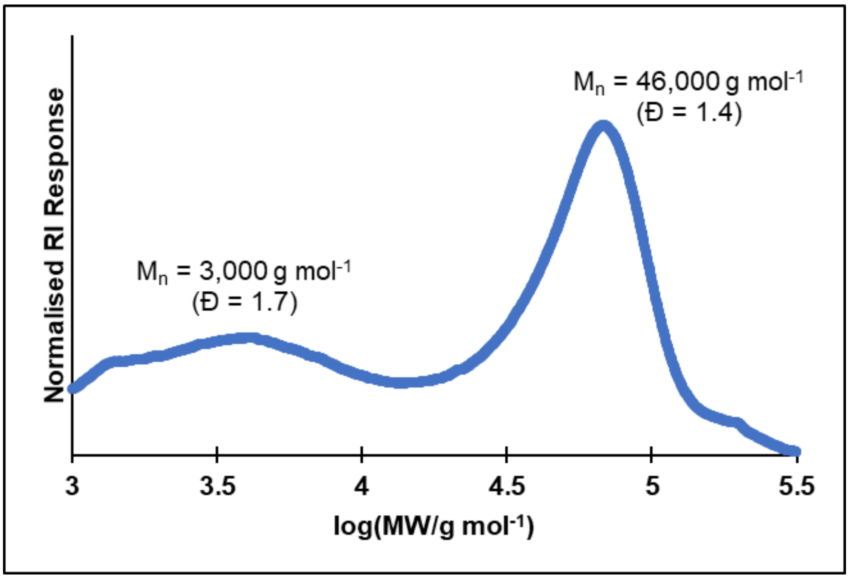

Figure S41. GPC trace of **BCP1**.

^{1}H DOSY NMR experiments

A ^{1}H-DOSY NMR experiment analysing **BCP1** shows that all of the signals diffuse at very similar rates at 2.0-2.24x 10^{-10} m/s. In comparison, a sample containing separate homopolymers of $[H_2BPPhH]_n$ and $[H_2BP(n\text{-}hex)H]_n$, was analysed by ^{1}H DOSY NMR and the two homopolymers clearly diffuse at different rates, at 1.5 x 10^{-10} m/s and 3.9 x 10^{-10} m/s respectively. Similar polymer molecular weights were used for all the polymers: **BCP1**; M_n = 40,500 g mol^{-1} $[H_2BPPhH]_n$; M_n = 44,000 g mol^{-1} and $[H_2BP(n\text{-}hex)H]_n$; M_n = 33,000 g mol^{-1}.

The apparent hydrodynamic radius (or Stokes radius) can be calculated from the measured diffusion coefficient as:

$$R_{H,app} = \frac{k_B T}{6\pi\eta D}$$

Where η is the viscosity. Treating the viscosity of $CDCl_3$ as 5.28 x10^{-4} kg m^{-1} s^{-1}, we find:

Table S5. Diffusion rate and apparent hydrodynamic radii of the homopolymer and copolymer samples determined by quantitative ^{1}H DOSY experiments.

	**Diffusion rate (x10^{-10} m^2 s^{-1})**	**Apparent hydrodynamic radius $R_{H,\,app}$ (nm)**
**BCP1**	1.95 ± 0.05	2.12 ± 0.05
$[H_2BPPhH]_n$	1.5 ± 0.2	2.8 ± 0.4
$[H_2BP(n\text{-}hex)H]_n$	3.9 ± 0.8	1.0 ± 0.3

There are several caveats to these R_H measurements. Firstly, they are not the same as the true hydrodynamic radius, although they are related to it by an unknown numerical factor which is different for each polymer. In addition, the polymers may increase the viscosity of each sample relative to pure $CDCl_3$: this would lead us to overestimate R_H. That aside, all polymers have similar values of $R_{H,app}$, suggesting these polymers do not form large micelles in $CDCl_3$.

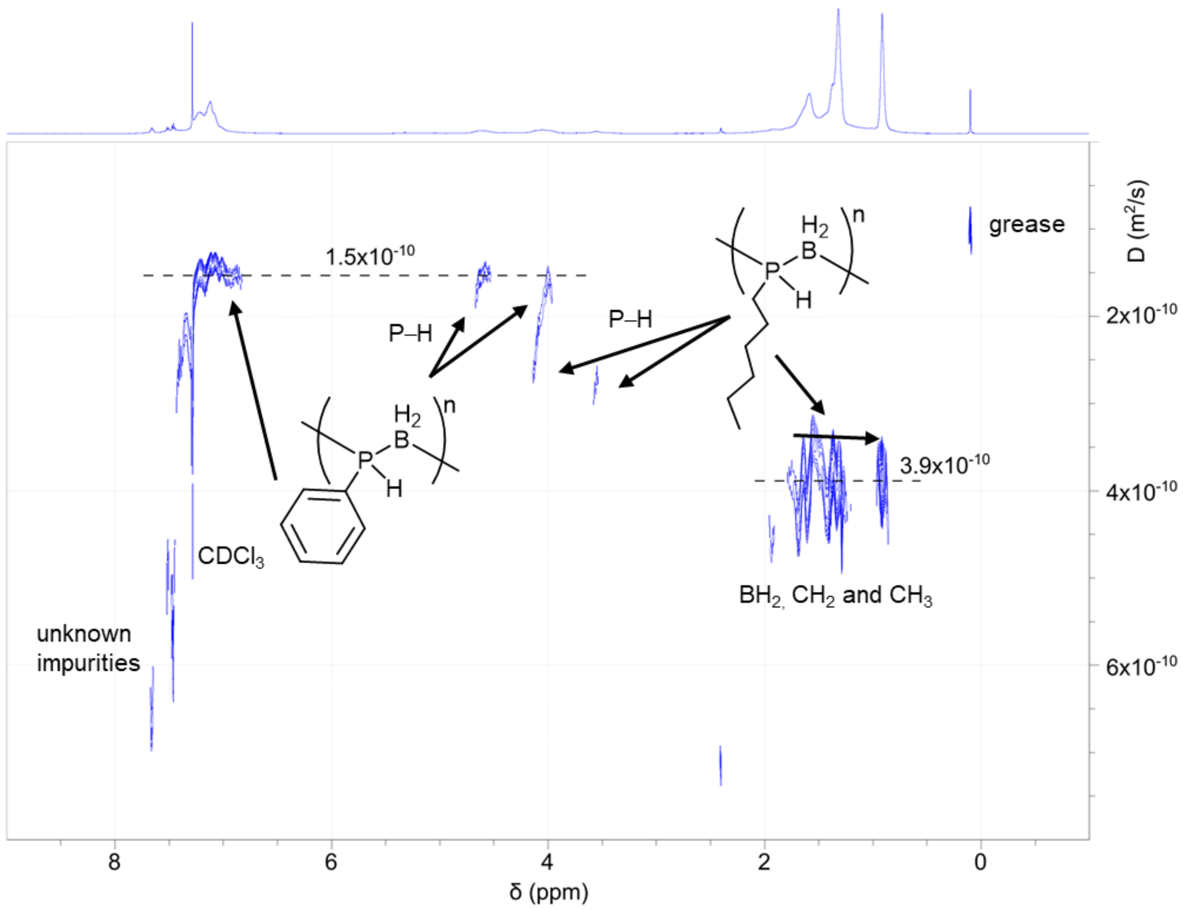

Figure S42. ^1H DOSY NMR spectrum of a mixed sample of the two homopolymers $[H_2BPPhH]_n$ and $[H_2BP(n\text{-hex})H]_n$ (600 MHz, $CDCl_3$, 298 K).

For **BCP1** There is a slight discrepancy in the measured diffusion coefficient s between the aromatic and aliphatic regions. To investigate further, the plots of signal intensity vs gradient strength in **BCP1** were examined at several different chemical shifts. If a single diffusing species is present, the signal intensity should vary as:

$$I = I_0 e^{-D\gamma^2 g^2 \delta^2 (\Delta - \delta/3)}$$

where I = signal intensity, I_0 is the initial signal intensity, D is the diffusion coefficient, γ is the gyromagnetic ratio, g is the gradient strength, δ is the gradient pulse length and Δ is the diffusion delay. If several species are present, the signal intensity is the sum of their individual signals:

$$I = \sum_i I_{0,i} e^{-D_i \gamma^2 g^2 \delta^2 (\Delta - \delta/3)}$$

In the aromatic region, the data fits well to the single species model (Figure S43B). In the aliphatic region however, large deviations are seen at high gradient strengths, indicating the presence of multiple species (Figure S43C). A two species model fits the data from the aliphatic region more closely (Figure S43D). The two species model also suggests that the slower diffusing species has a diffusion coefficient of approximately $2.0x10^{-10}$ m^2/s, in good agreement with the aromatic region. This suggests the sample contains one copolymer with diffusion coefficient of $2.0x10^{-10}$ m^2/s, and one or more smaller species with signals in the aliphatic region.

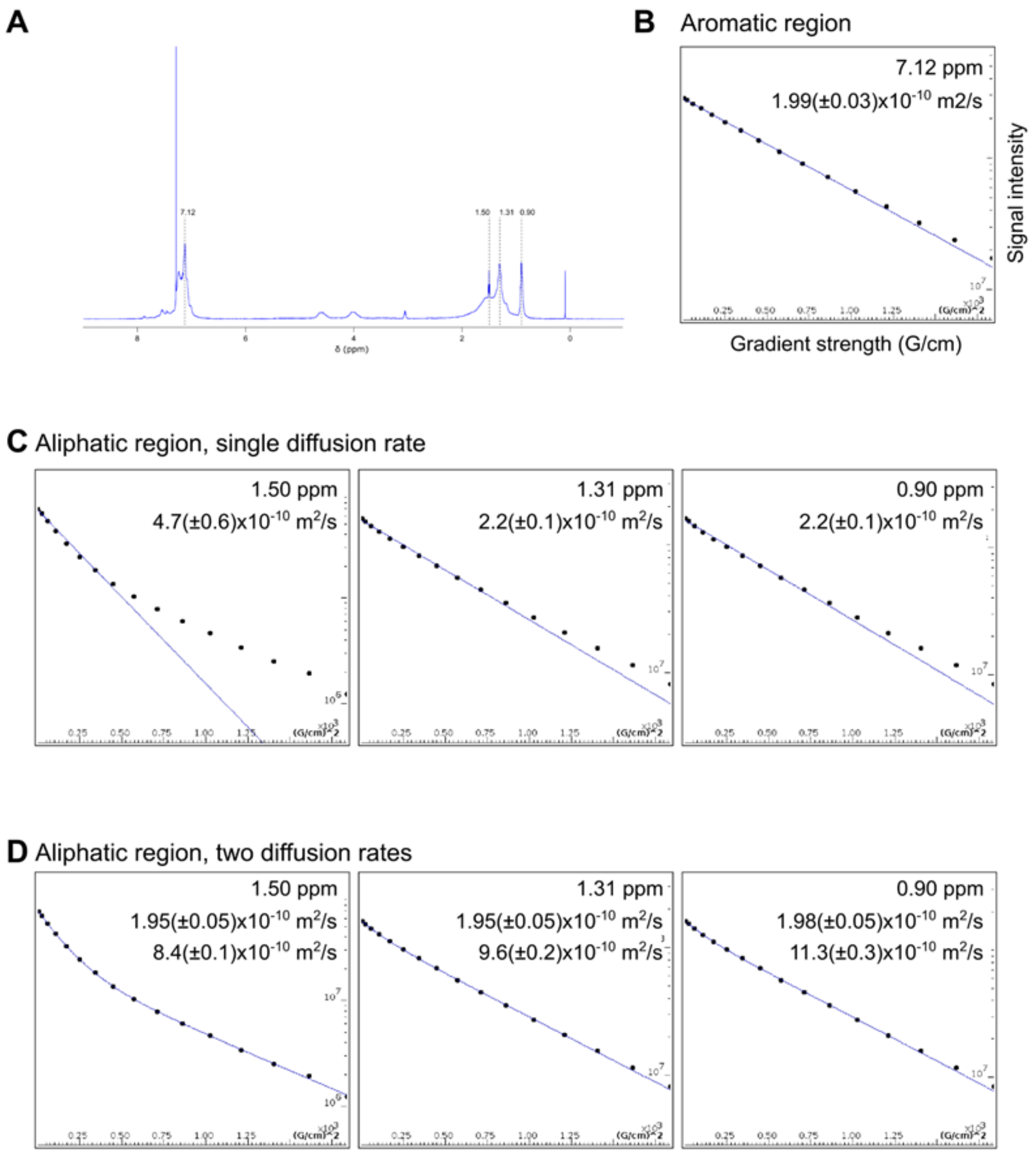

Figure S43. **A**: ^{1}H NMR spectrum of **BCP1** showing the peaks used for diffusion fitting. **B-D**: Fitting was performed using peak intensity at the given chemical shifts using Bruker Dynamics Centre. Data was fit to models with either one or two terms of the form: $= I_0 e^{-D\gamma^2 g^2 \delta^2 (\Delta - \delta/3)}$. The fitted diffusion coefficients and 95% confidence intervals are shown in the top right of each plot. **B**: Fit of a single diffusion coefficient in the aromatic region. **C**: Fits of single diffusion coefficients to peaks in the aliphatic region. **D**: Fits of a 2-term diffusion model to peaks in the aliphatic region.

Dynamic Light Scattering

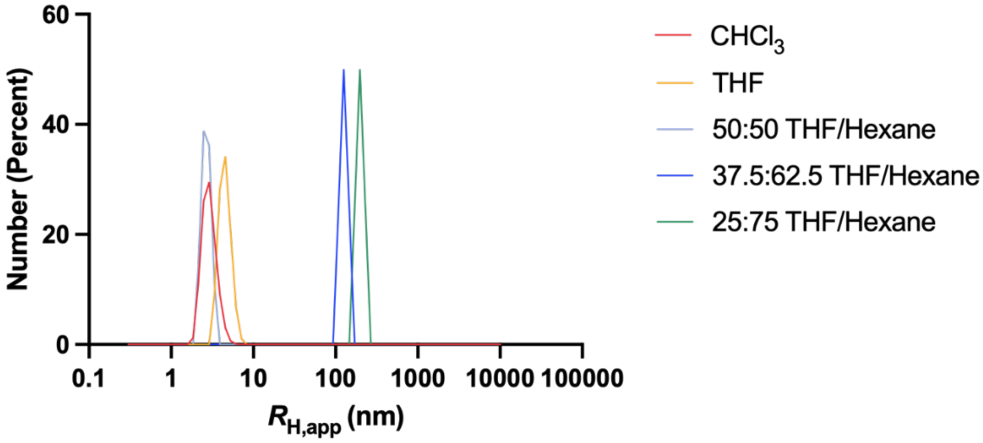

Figure S44. DLS number plots of 0.1 mg•mL^{-1} solutions of **BCP1** in $CHCl_3$, THF, and in THF/hexane mixtures.

Table S6. Calculated hydrodynamic radii of **BCP1** in different solvents by DLS.

Solvent	$R_{H,app}$ (nm)
$CHCl_3$	2.7 ± 2.0
THF	3.0 ± 1.4
50:50 THF:hexane	3.3 ± 1.7
37.5:62.5 THF:hexane	132 ± 12
25:75 THF:hexane	180 ± 30

Transmission electron microscopy

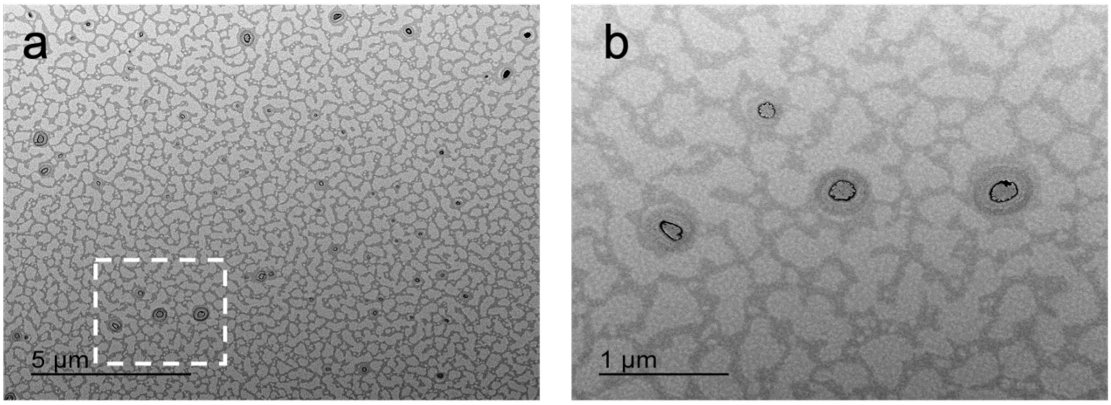

Figure S45. TEM images of assembled structures of $[PhPHBH_2]_{110}$-b-$[H_2BP(n\text{-hexyl})]_{11}H$, **BCP1**, obtained from a 25%:75% v/v THF:hexane solution, without staining and after allowing the solvent to evaporate. The structures highlighted in the box in **a** are shown with a fivefold magnification in **b**. The carbon film substrate also shows evidence for the deposition of a unimer film formed by residual molecularly dissolved **BCP1**.

The approximate concentration of residual Rh in **BCP1** was determined using inductively coupled plasma mass spectrometry (ICP-MS) techniques on an Agilent 8800 triple quadrupole ICP-MS instrument. For sample preparation, 1 mg of **BCP1** was digested in 1 mL of refluxing environmental grade nitric acid overnight (16 h). The resulting digested sample was diluted by a factor of 1 in 10,000 to an approximate 100 $ng_{\mathbf{BCP1}}$•mL^{-1} concentration using an 2% v/v nitric acid solution in water and subsequently analyzed. It was found that there was approximately 1 ng_{Rh}•mL^{-1} (1 ppm) in the sample. Additionally, two procedural blanks without **BCP1** were also prepared in an identical manner and no Rh was detected. Considering these samples lack an internal standard, the determined Rh concentration should be considered approximate.

References

[1] G. Giordano, R. H. Crabtree, R. M. Heintz, D. Forster, D. E. Morris. *Inorg. Synth.,* **1990,** 88-90.
[2] K. Bourumeau, A.-C. Gaumont, J.-M. Denis. *J. Organomet. Chem.,* **1997,** 529, 205-213.
[3] J. R. Turner, D. A. Resendiz-Lara, T. Jurca, A. Schäfer, J. R. Vance, et al., *Macromol. Chem. Phys.,* **2017,** 218, 1700120.
[4] H. Cavaye, F. Clegg, P. J. Gould, M. K. Ladyman, T. Temple, et al., *Macromolecules,* **2017,** 50, 9239-9248.
[5] A. T. Lubben, J. S. McIndoe, A. S. Weller. *Organometallics,* **2008,** 27, 3303-3306.
[6] H. Dorn, R. A. Singh, J. A. Massey, A. J. Lough, I. Manners. *Angew. Chem. Int. Ed.,* **1999,** 38, 3321-3323.
[7] D. A. Resendiz-Lara, V. T. Annibale, A. W. Knights, S. S. Chitnis, I. Manners. *Macromolecules,* **2021,** 54, 71-82.
[8] N. L. Oldroyd, S. S. Chitnis, V. T. Annibale, M. I. Arz, H. A. Sparkes, et al., *Nature Communications,* **2019,** 10, 1370.
[9] T. N. Hooper, A. S. Weller, N. A. Beattie, S. A. Macgregor. *Chem. Sci.,* **2016,** 7, 2414-2426.
[10] T. N. Hooper, M. A. Huertos, T. Jurca, S. D. Pike, A. S. Weller, et al., *Inorg. Chem.,* **2014,** 53, 3716-3729.
[11] M. A. Huertos, A. S. Weller. *Chem. Sci.,* **2013,** 4, 1881-1888.
[12] R. Dallanegra, A. P. M. Robertson, A. B. Chaplin, I. Manners, A. S. Weller. *ChemComm.,* **2011,** 47, 3763-3765.
[13] M. Mizutani, K. Satoh, M. Kamigaito. *J. Am. Chem. Soc.,* **2010,** 132, 7498-7507.

[14] J. Stetefeld, S. A. McKenna, T. R. Patel. *Biophys. Rev.,* **2016,** 8, 409-427.
